# Computational biology and in vitro studies for anticipating cancer-related molecular targets of sweet wormwood (*Artemisia annua*)

**DOI:** 10.1186/s12906-023-04135-0

**Published:** 2023-09-08

**Authors:** Hend Dawood, Ismail Celik, Reham S. Ibrahim

**Affiliations:** 1 Department of Pharmacognosy, Faculty of Pharmacy, Alexandria University, Alexandria, 21521 Egypt; 2 Department of Pharmaceutical Chemistry, Faculty of Pharmacy, Erciyes University, Kayseri, 38039 Turkey

**Keywords:** *Artemisia annua*, Molecular docking, Dynamic simulation, Cancer, Network pharmacology

## Abstract

**Background:**

Cancer is one of the leading causes of death worldwide. Recently, it was shown that many natural extracts have positive effects against cancer, compared with chemotherapy or recent hormonal treatments. *A. annua* is an annual medicinal herb used in the traditional Chinese medicine. It has also been shown to inhibit the proliferation of various cancer cell lines.

**Methods:**

Multi-level modes of action of *A. annua* constituents in cancer therapy were investigated using an integrated approach of network pharmacology, molecular docking, dynamic simulations and *in-vitro* cytotoxicity testing on both healthy and cancer cells.

**Results:**

Network pharmacology-based analysis showed that the hit *Artemisia annua* constituents related to cancer targets were 3-(2-methylpropanoyl)-4-cadinene-3,11-diol, artemisinin G, O-(2-propenal) coniferaldehyde, (2-glyceryl)-O-coniferaldehyde and arteamisinin III, whereas the main cancer allied targets were NFKB1, MAP2K1 and AR. Sixty-eight significant signaling KEGG pathways with *p* < 0.01 were recognized, the most enriched of which were prostate cancer, breast cancer, melanoma and pancreatic cancer. Thirty-five biological processes were mainly regulated by cancer, involving cellular response to mechanical stimulus, positive regulation of gene expression and transcription. Molecular docking analysis of the top hit compounds against the most enriched target proteins showed that 3-(2-methylpropanoyl)-4-cadinene-3,11-diol and O-(2-propenal) coniferaldehyde exhibited the most stabilized interactions. Molecular dynamics simulations were performed to explain the stability of these two compounds in their protein-ligand complexes. Finally, confirmation of the potential anticancer activity was attained by *in-vitro* cytotoxicity testing of the extract on human prostate (PC-3), breast (MDA-MB-231), pancreatic (PANC-1) and melanoma (A375) cancerous cell lines.

**Conclusion:**

This study presents deeper insights into *A. annua* molecular mechanisms of action in cancer for the first time using an integrated approaches verifying the herb’s value.

**Supplementary Information:**

The online version contains supplementary material available at 10.1186/s12906-023-04135-0.

## Introduction

Cancer is an essential health problem and one of the most leading causes of death worldwide [1]. Carcinogenesis is a multistage process that initiates with genetic alteration due to the activation of oncogenes and the inactivation of tumor suppressor genes resulting in a loss of control of cell proliferation [2]. The alteration of normal cells into malignant ones is regulated by several steps, each may serve as a target for anticancer agents [3].

Prevention of cancer is one of the most crucial medicinal issues in the past few years [4]. Recently, many natural products have been studied for their chemo-preventive effects. It has been shown that many extracts from natural products have positive effects against cancer, compared with chemotherapy or recent hormonal treatments due to their availability, lower side effects and cost effectiveness [5]. Hence, they form approximately 60% of all anti-cancer drugs and have captivated scientific attention as promising anti-cancer therapies. The assessment of traditional phytomedicines for cytotoxic activity may contribute to the innovation of new chemotherapeutic regimens either alone or combined with the existing chemotherapeutic drugs so that to combat various cancer types [6]. Cytotoxicity of these phytomedicines arises from their ability to suppress certain tumor cell characteristics, such as increased metabolism, elevated concentration of iron and transferrin, and susceptibility to Reactive Oxygen Species (ROS) [7].

*Artemisia annua* (family Asteraceae) also known as ‘Sweet wormwood’ (English) or ‘Qinghao’ (Chinese), is an annual medicinal herb naturally grown in China, with a long history in treating various diseases in the traditional Chinese medicine including fever, amoebiasis, cancer, schistosomiasis, HIV and Leishmaniasis [8]. This plant is known for its aromatic scent, bitter taste, and aromatic pale yellow blossoms [9]. Beside its promising anti-inflammatory, antimalarial and immunosuppressive activities, it has also been shown to inhibit the proliferation of various cancer cell lines. Sesquiterpene lactones as artemisinin were identified as important active principles of *Artemisia annua* extract, however, available evidence assumed that artemisinin might not be the most active anticancer ingredient of this medicinal plant. Accordingly, *Artemisia annua* may serve as a source for new anti-cancer components [10] as it is enriched with phenolic compounds with high antioxidant power classified into mainly five major groups; coumarin, flavones, flavonols and phenolic acids [9]. Up to our knowledge, few studies have explained the potential role of these metabolites and their synergistic effects in the treatment of cancer, thus, the molecular mechanisms for the anti-cancer activities of *Artemisia annua* still need elucidation.

Owing to the multi-chemical components of the plant extracts, their multi-pharmacological effects as well as their multi-action targets in the treatment of different diseases, it is challenging for traditional methods to demonstrate the different mechanisms behind their pharmacological activities in an adequate manner [11, 12]. Yet, a recently emerging approach named network pharmacology had combined bioinformatics and pharmacology organically thus can clarify and explain the multi-mechanisms of action of these plants via emphasizing the relationship between targets, components and diseases [13, 14]. Accordingly, this approach was capable of affording new ideas for research and development of herbal medicine [15].

In this study, we aimed to overview the material basis and the detailed mechanism of *Artemisia annua* in the treatment of different cancer types and highlight the specific type of cancer cells that are mostly inhibited for their proliferation using network pharmacology analysis. The interaction between the most enriched compounds and bio-macromolecule targets was illustrated by molecular docking to estimate the binding patterns and affinity. However, network pharmacology and molecular docking are just in-silico techniques that provide a simulation model but cannot reflect realistic complexity, accordingly, *in-vitro* cytotoxicity experiments against the top hit cancer cells – retrieved from network pharmacology- were conducted to verify and provide complementary information. Ultimately, the study in hand could provide a new perceptiveness and guidance for the development of *Artemisia annua* in cancer treatment.

## Results and discussion

### Network pharmacology analysis

**Fig. 1:**
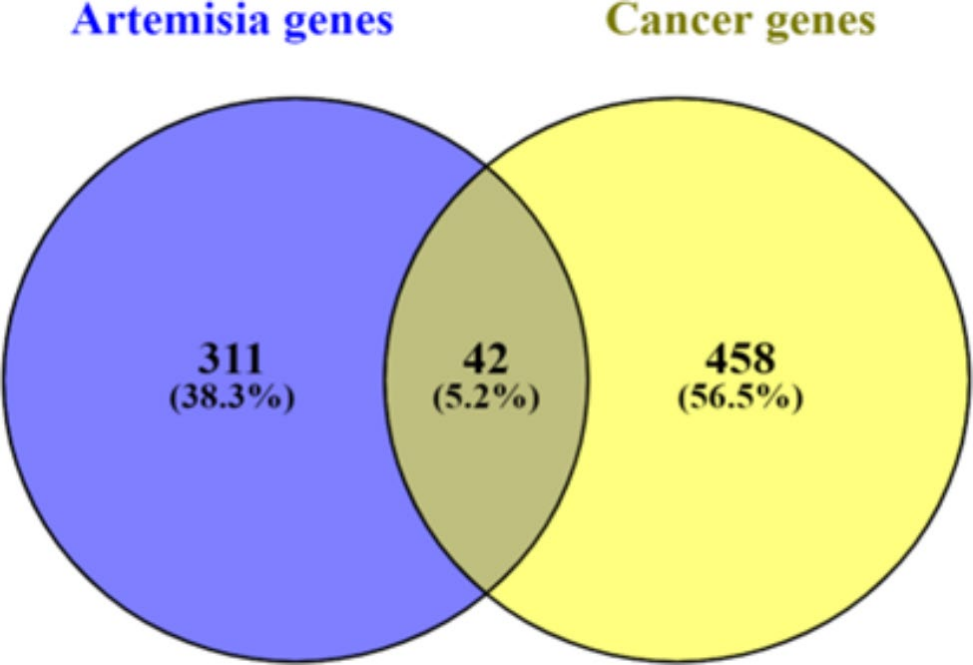
Venn diagram of *Artemisia annua* compounds and cancer targets

From the dictionary of natural products database, 80 *Artemisia annua* compounds were identified while, their predicted targets were collected from the Swiss Target Prediction platform, generating a total of 340 targets. 487 cancer-related target genes were yielded from the GeneCards (Table S1), and 29 intersecting druggable target genes of the corresponding active ingredients of *Artemisia annua* were identified by mapping the compounds’ genes to the cancer-associated genes (Fig. 1).

**Fig. 2:**
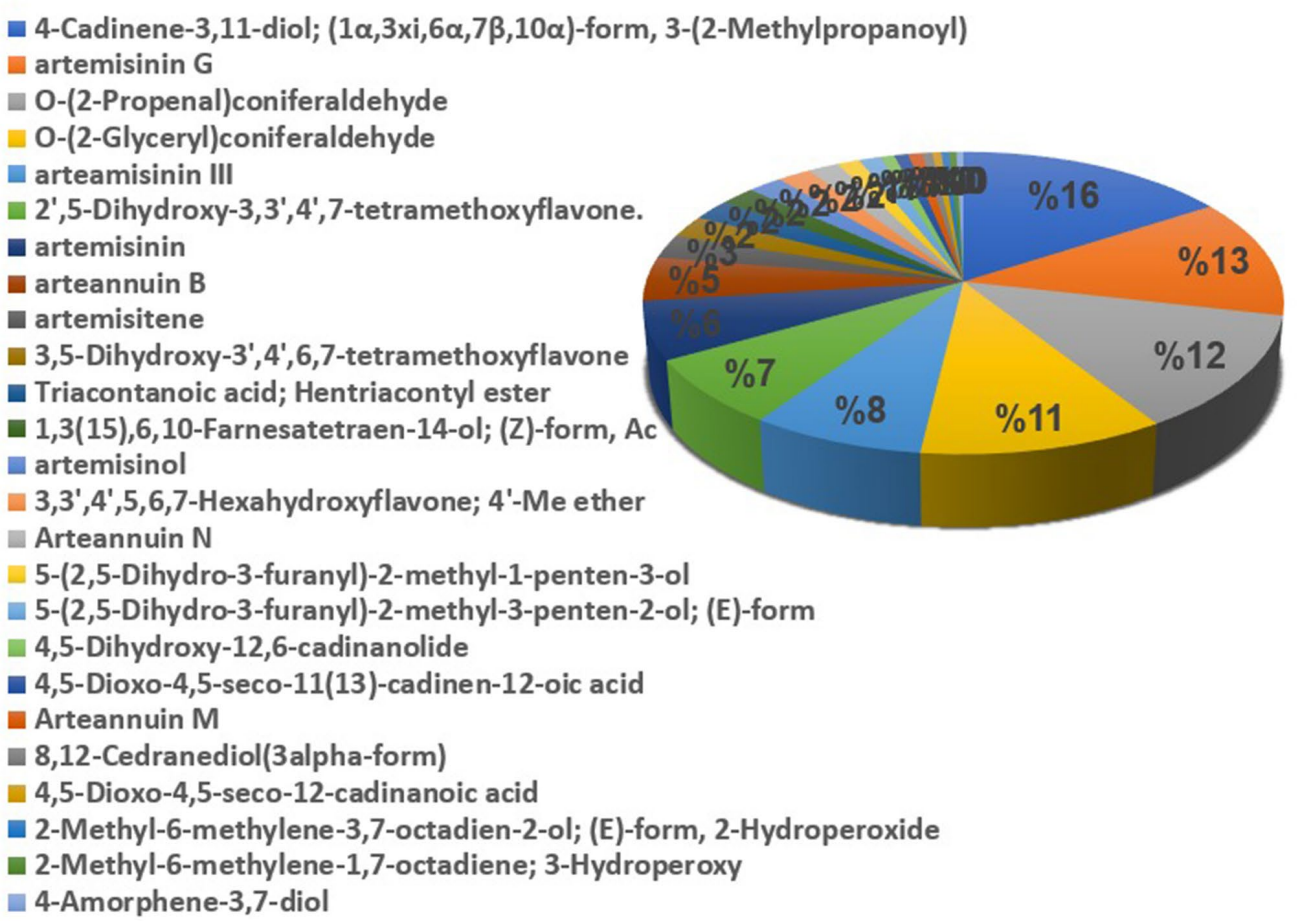
The distributions % of *Artemisia annua* L. constituents’ interactions with 29 intersecting cancer-related target genes

It is worth mentioning that 25 compounds out of 80 *Artemisia annua* metabolites showed interactions with cancer-related targets. Based on the compounds’ predicted interaction probabilities retrieved from Swiss Target Prediction database, they contributed differently to the pharmacological profile of the plant where, 4-Cadinene-3,11-diol; (1α,3xi,6α,7β,10α)-form, 3-(2-Methylpropanoyl) accounted for 16% of the total interactions followed by artemisinin G, O-(2-Propenal)coniferaldehyde and O-(2-Glyceryl) coniferaldehyde contributing with 13%, 12% and 11% respectively of the total interactions (Fig. 2). This revealed that these compounds might be the core metabolites for the anti-cancer activity of *Artemisia annua*.

**Fig. 3:**
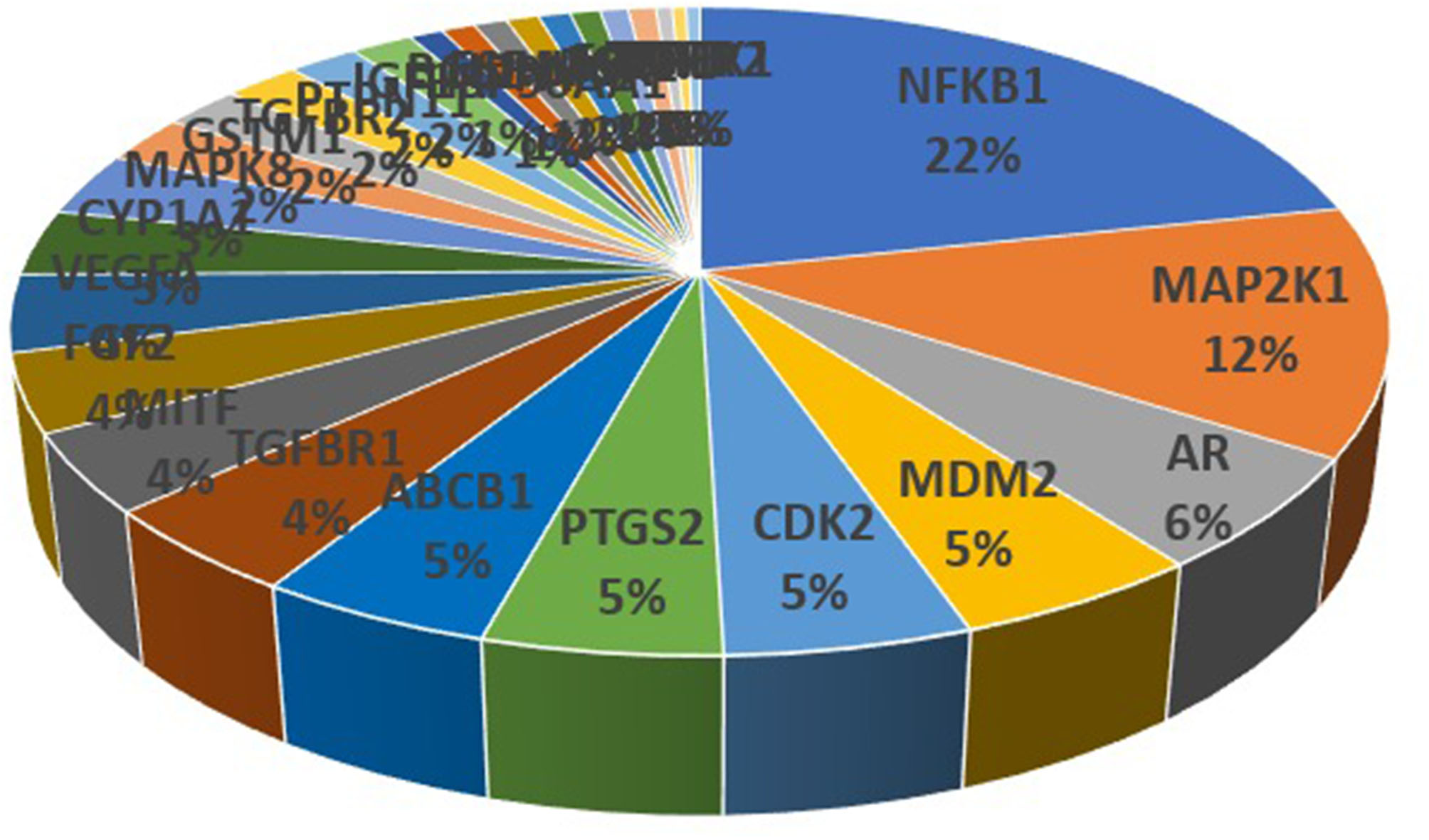
The distributions % of target genes interactions with *Artemisia annua* L. constituents

As far as it can be noted, this plant appeared to exhibit its therapeutic effect on cancer by regulating these 29 intersecting target genes, among them, Nuclear factor NF-kappa-B p105 subunit (NFKB1), mitogen-activated protein kinase kinase 1 (MAP2K1), Androgen Receptor (AR) and p53-binding protein (MDM2) had the highest sum of interaction probability scores as reflected by their percentages of interactions (22%, 12%, 6% and 5%, respectively) (Fig. 3; Table 1). This indicated their valuable contribution as therapeutic cancer targets on which *Artemisia annua* could act. Cancer is regulated by NFKB1 gene as indicated by the notion that genetic variants of *NFKB1* might increase the susceptibility of liver [16] and gastric cancer [17]. Moreover, targeting NFKB1/RELA interaction could mitigate Ets1-mediated metastatic breast cancer [18]. Not only NFKB1, MAP2K1 is one of the target proteins in cancer pathogenesis where, a previous study proved that miRNAs repressed the carcinogenesis of the human non-small cell lung cancer (NSCLC) by inhibiting MAP2K1 expression of MAP2K1 via direct targeting its 3’UTR [19]. Furthermore, *MAP2K1* mutation lead to poor response to EGFR inhibition and increase pathogenesis of colorectal cancer [20] and thyroid gland cancer [21]. AR inhibitors are regarded as promising agents in cancer treatment as they decreased IL-6 production by breast cancer cells (MB-231 and MB-231BR) so inhibit their proliferation [22]. In addition, inhibition of AR diminished the synthesis of androgens in prostate cancer [23]. MDM2 protein also takes a significant part in the pathology of cancer in which, MDM2 inhibitors reactivated p53 and treated some types of cancer as Malignant pleural mesothelioma (MPM) [24]. Furthermore, targeting over-expressed MDM2 interrupted membrane integrity by pore formation, triggered membrane destabilization and rapid cancer cell necrosis [25].

**Table 1:** Potential protein targets of *Artemisia annua* constituents

Target protein short name	Full name of protein	Uniprot accession no.	Interacting compounds (interaction probability Score)
Nuclear factor NF-kappa-B p105 subunit	NFKB1	P19838	O-(2-Glyceryl)coniferaldehyde (0.43), O-(2-Propenal)coniferaldehyde (0.5)
Dual specificity mitogen-activated protein kinase kinase 1	MAP2K1	Q02750	artemisinin G (0.1005), artemisinin III (0.112)
Androgen Receptor	AR	P10275	1,3(15),6,10-Farnesatetraen-14-ol; (Z)-form, Ac (0.112), 4,5-Dioxo-4,5-seco-11(13)-cadinen-12-oic acid (0.112), 4,5-Dioxo-4,5-seco-12-cadinanoic acid 4-Cadinene-3,11-diol; (1&alpha;,3&xi;,6&alpha;,7&beta;,10&alpha;)-form, 3-(2-Methylpropanoyl), 5-(2,5-Dihydro-3-furanyl)-2-methyl-1-penten-3-ol, 8,12-Cedranediol(3α-form), Arteannuin N, artemisinin G, artemisinol, 4-Amorphene-3,7-diol
p53-binding protein Mdm-2	MDM2	Q00987	4-Cadinene-3,11-diol; (1α,3xi,6α,7β,10β)-form, 3-(2-Methylpropanoyl) (0.1115), artemisinin G (0.1005)
Cyclin-dependent kinase 2	CDK2	P24941	Artemisinin (0.1005), artemisinin III (0.112), artemisitene (0.1005)
Cyclooxygenase-2	PTGS2	P35354	4,5-Dihydroxy-12,6-cadinanolide (0.112), 4-Cadinene-3,11-diol; (1α,3xi,6α,7β,10β)-form, 3-(2-Methylpropanoyl) (0.1115), 5-(2,5-Dihydro-3-furanyl)-2-methyl-3-penten-2-ol; (E)-form (0.116), Arteannuin M (0.112)
ATP-dependent translocase ABCB1	ABCB1	P08183	3,3’,4’,5,6,7-Hexahydroxyflavone; 4’-Me ether (0.54), 3,5-Dihydroxy-3’,4’,6,7-tetramethoxyflavone (0.65)
TGF-beta receptor type I	TGFBR1	P37173	1,3(15),6,10-Farnesatetraen-14-ol; (Z)-form, Ac (0.112), 4-Cadinene-3,11-diol; (1α,3xi,6α,7β,10β)-form, 3-(2-Methylpropanoyl) (0.1115)
Microphthalmia-associated transcription factor	MITF	O75030	Arteannuin B (1.0)
Fibroblast growth factor 2	FGF2	P09038	2’,5-Dihydroxy-3,3’,4’,7-tetramethoxyflavone (0.33)
Vascular endothelial growth factor A	VEGFA	P15692	2’,5-Dihydroxy-3,3’,4’,7-tetramethoxyflavone (0.33)
Cytochrome P450 1A1	CYP1A1	P11511	O-(2-Glyceryl)coniferaldehyde (0.42), O-(2-Propenal)coniferaldehyde (0.43)
c-Jun N-terminal kinase 1	MAPK8	P45983	Artemisinin (0.1005), artemisinin G (0.1005), artemisitene (0.1005), 4-Cadinene-3,11-diol; (1α,3xi,6α,7β,10β)-form, 3-(2-Methylpropanoyl) (0.1115)
Glutathione S-transferase Mu 1	GSTM1	P09488	Triacontanoic acid; Hentriacontyl ester (0.31)
TGF-beta receptor type II	TGFBR2	P37173	4-Cadinene-3,11-diol; (1α,3xi,6α,7β,10β)-form, 3-(2-Methylpropanoyl) (0.1115)
Protein-tyrosine phosphatase 2 C	PTPN11	Q06124	Arteannuin N (0.162) dihydroartemisinic acid (0.112)
Insulin-like growth factor I receptor	IGF1R	P08069	artemisinin G (0.1005)
Apoptosis regulator Bcl-2	BCL2	P10415	4-Cadinene-3,11-diol; (1α,3xi,6α,7β,10β)-form, 3-(2-Methylpropanoyl) (0.1115)
Estrogen receptor alpha	ESR1	P03372	Artemisinol (0.242)
Heat shock protein HSP 90-alpha	HSP90AA1	P07900	2-Methyl-6-methylene-1,7-octadiene; 3-Hydroperoxy (0.116), 2-Methyl-6-methylene-3,7-octadien-2-ol; (E)-form, 2-Hydroperoxide (0.125)
Peroxisome proliferator-activated receptor gamma	PPARG	P37231	4,5-Dioxo-4,5-seco-11(13)-cadinen-12-oic acid (0.112), 4-Cadinene-3,11-diol; (1α,3xi,6α,7β,10β)-form, 3-(2-Methylpropanoyl) (0.127)
Hypoxia-inducible factor 1 alpha	HIF1A	Q16665	4-Cadinene-3,11-diol; (1α,3xi,6α,7β,10β)-form, 3-(2-Methylpropanoyl) (0.1115), 5-(2,5-Dihydro-3-furanyl)-2-methyl-1-penten-3-ol (0.125)
Progesterone receptor	PGR	P06401	5-(2,5-Dihydro-3-furanyl)-2-methyl-1-penten-3-ol (0.117), 4-Cadinene-3,11-diol; (1α,3xi,6α,7β,10β)-form, 3-(2-Methylpropanoyl) (0.1115)
Estrogen receptor beta	ESR2	Q92731	5-(2,5-Dihydro-3-furanyl)-2-methyl-3-penten-2-ol; (E)-form (0.116), artemisinin G (0.1005)
Fibroblast growth factor receptor 1	FGFR1	P11362	artemisinin G (0.1005)
Telomerase reverse transcriptase	TERT	O14746	artemisinin G (0.1005)
Epidermal growth factor receptor erbB1	EGFR	Q9WTS1	Artemisinin (0.1005), 4-Cadinene-3,11-diol; (1α,3xi,6α,7β,10β)-form, 3-(2-Methylpropanoyl) (0.1115)
Matrix metalloproteinase 2	MMP2	P08253	artemisinin G (0.1005)
Serine/threonine-protein kinase Chk1	CHEK1	O14757	Artemisitene (0.1005)

**Fig. 4:**
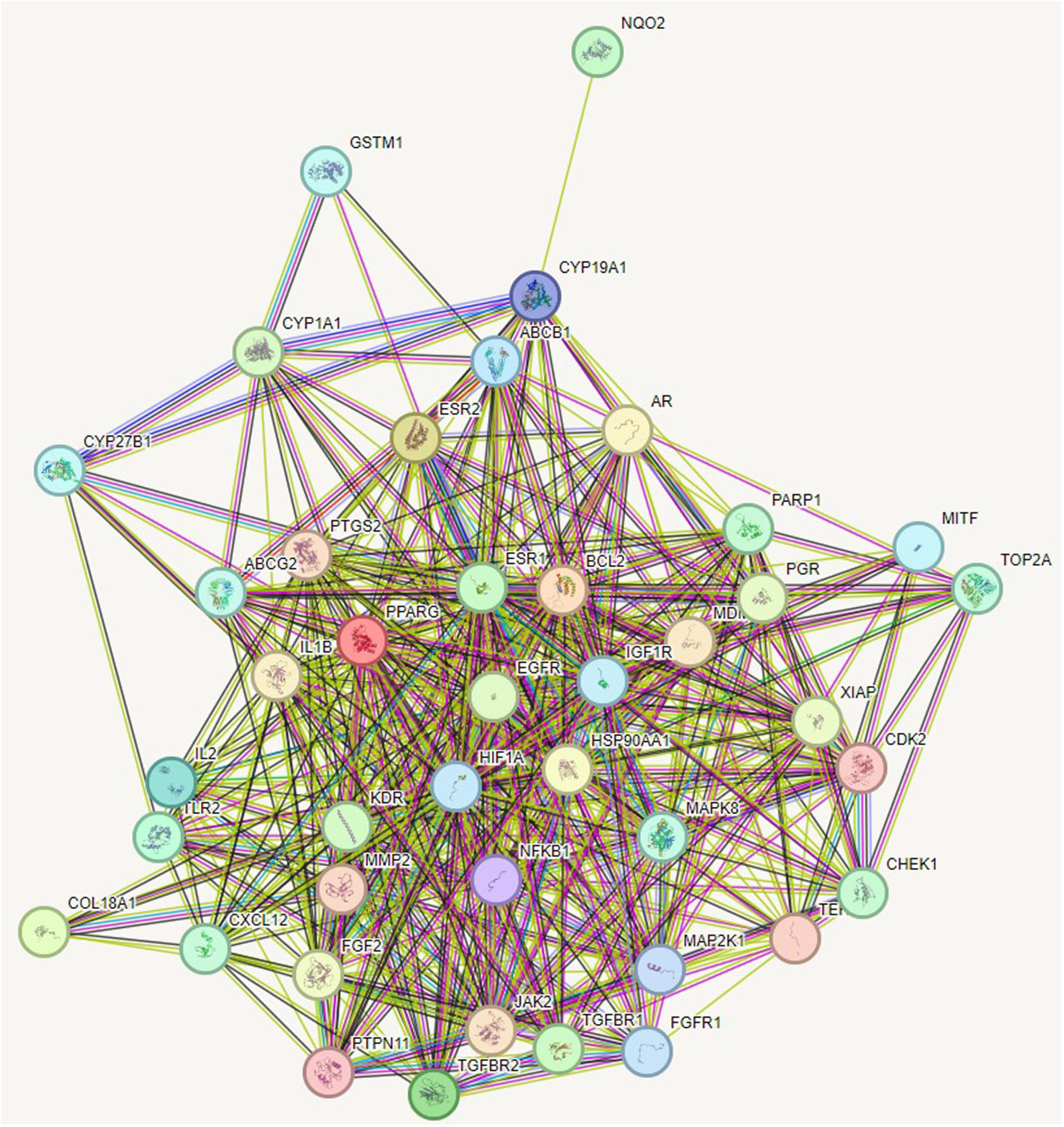
Protein-Protein interaction diagram of *Artemisia annua*

From STRING analysis, the overlapping genes were closely associated in the protein-protein interaction diagram (Fig. 4, Table S2).

Based on the aforementioned 29 intersecting target genes, 68 significant signaling KEGG pathways with *p* < 0.01 were generated, from which, the most enriched 17 cancer – related pathways were picked up for further analysis including Prostate cancer, breast cancer, melanoma and pancreatic cancer. As can be observed (Table 2), prostate cancer was recognized as the crucial key pathway in cancer with lowest *p* value (4.06E-12) and highest observed gene count [10].

**Table 2:** KEGG pathway analysis of potential target genes functions

#term ID	term description	false discovery rate (*p* value)	matching proteins in your network (labels)	observed gene count
hsa05215	Prostate cancer	4.06E-12	NFKB1,MDM2,CDK2,IGF1R,EGFR,MAP2K1,HSP90AA1,AR,BCL2,FGFR1	10
hsa05224	Breast cancer	3.92E-08	FGF2,IGF1R,EGFR,MAP2K1,PGR,ESR2,FGFR1,ESR1	8
hsa05218	Melanoma	1.35E-08	MDM2,FGF2,IGF1R,EGFR,MITF,MAP2K1,FGFR1	7
hsa05212	Pancreatic cancer	1.40E-08	NFKB1,EGFR,MAP2K1,TGFBR2,TGFBR1,MAPK8,VEGFA	7
hsa05225	Hepatocellular carcinoma	1.11E-06	IGF1R,EGFR,MAP2K1,TERT,GSTM1,TGFBR2,TGFBR1	7
hsa05220	Chronic myeloid leukemia	3.81E-07	NFKB1,MDM2,MAP2K1,PTPN11,TGFBR2,TGFBR1	6
hsa05210	Colorectal cancer	5.77E-07	EGFR,MAP2K1,TGFBR2,TGFBR1,MAPK8,BCL2	6
hsa05219	Bladder cancer	7.15E-07	MMP2,MDM2,EGFR,MAP2K1,VEGFA	5
hsa05222	Small cell lung cancer	1.98E-05	NFKB1,CDK2,PTGS2,XIAP,BCL2	5
hsa05211	Renal cell carcinoma	0.00012	MAP2K1,PTPN11,HIF1A,VEGFA	4
hsa05214	Glioma	0.00015	MDM2,IGF1R,EGFR,MAP2K1	4
hsa05203	Viral carcinogenesis	0.0036	NFKB1,MDM2,CDK2,CHEK1	4
hsa05204	Chemical carcinogenesis	0.0031	GSTM1, PTGS2, CYP1A1	3
hsa05216	Thyroid cancer	0.0119	PPARG, MAP2K1	2
hsa05213	Endometrial cancer	0.0251	EGFR, MAP2K1	2
hsa05221	Acute myeloid leukemia	0.0316	NFKB1, MAP2K1	2
hsa05223	Non-small cell lung cancer	0.0329	EGFR, MAP2K1	2

**Fig. 5:**
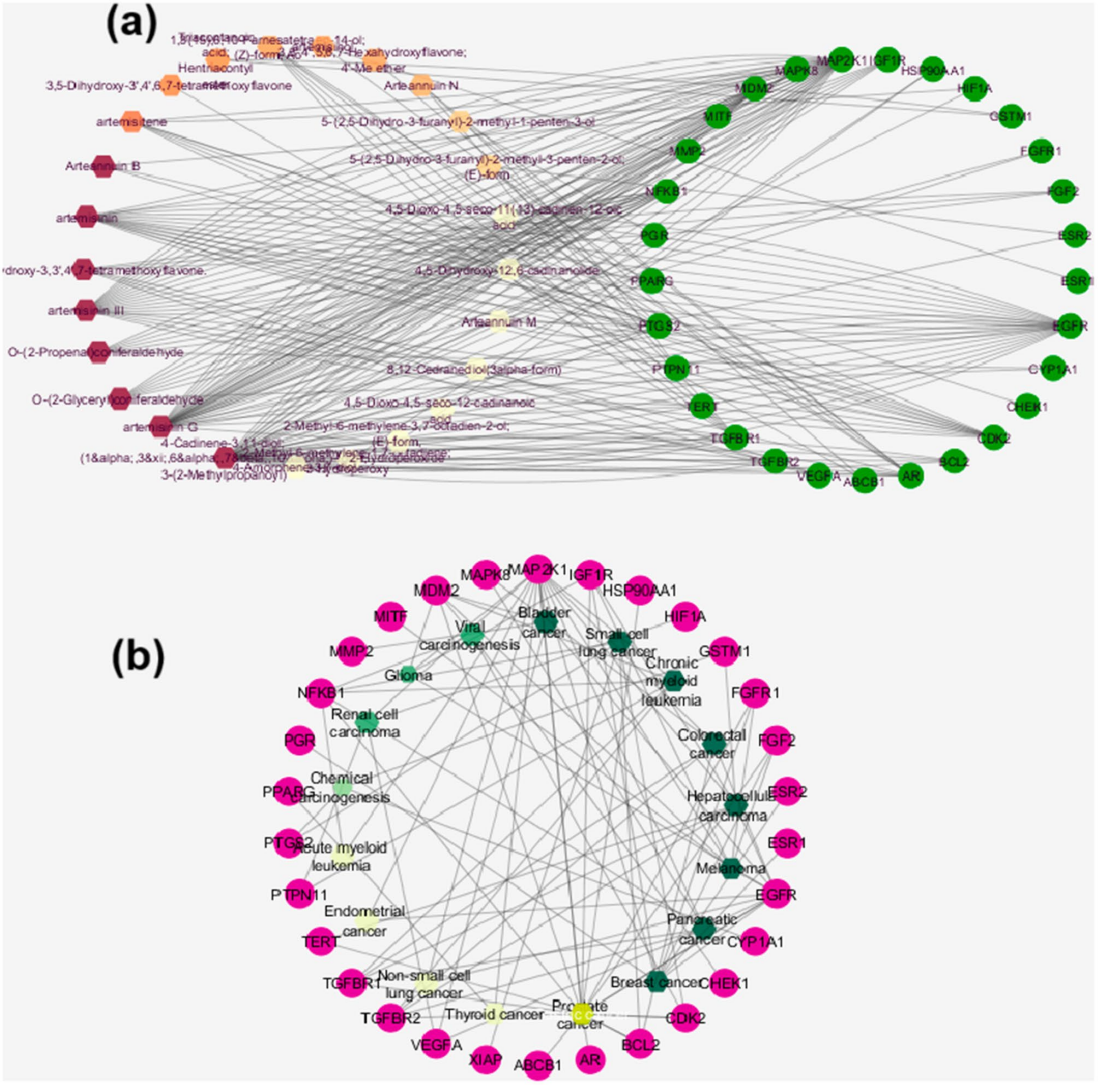
(**a**) Compounds-targets network. (**b**) Genes-pathway network. The nodes represent targets (green circles), compounds (red hexagons) and pathways (green hexagons). The edges were regarded as the association between the nodes. Enrichment of nodes are indicated by different color intensities

Cytoscape software was used to build “component-target’’ and ‘’ gene-pathway” networks (Fig. 5a and b). As can be observed, *Artemisia annua* related multi-component, multi-target, and multi-pathway mechanisms for cancer treatment. Figure (5a) showed interactions between components and target genes. The network included 55 nodes (sorted as 25 components and 29 genes) and 310 edges. It also suggested that not only the single component acted on multiple targets but also the same target was hit by different components. Similarly, KEGG pathway annotation revealed that the 29 potential target genes were involved in 18 significantly cancer - correlated pathways (Fig. 5b).

### KEGG pathway and gene ontology (GO) enrichment analysis

**Fig. 6:**
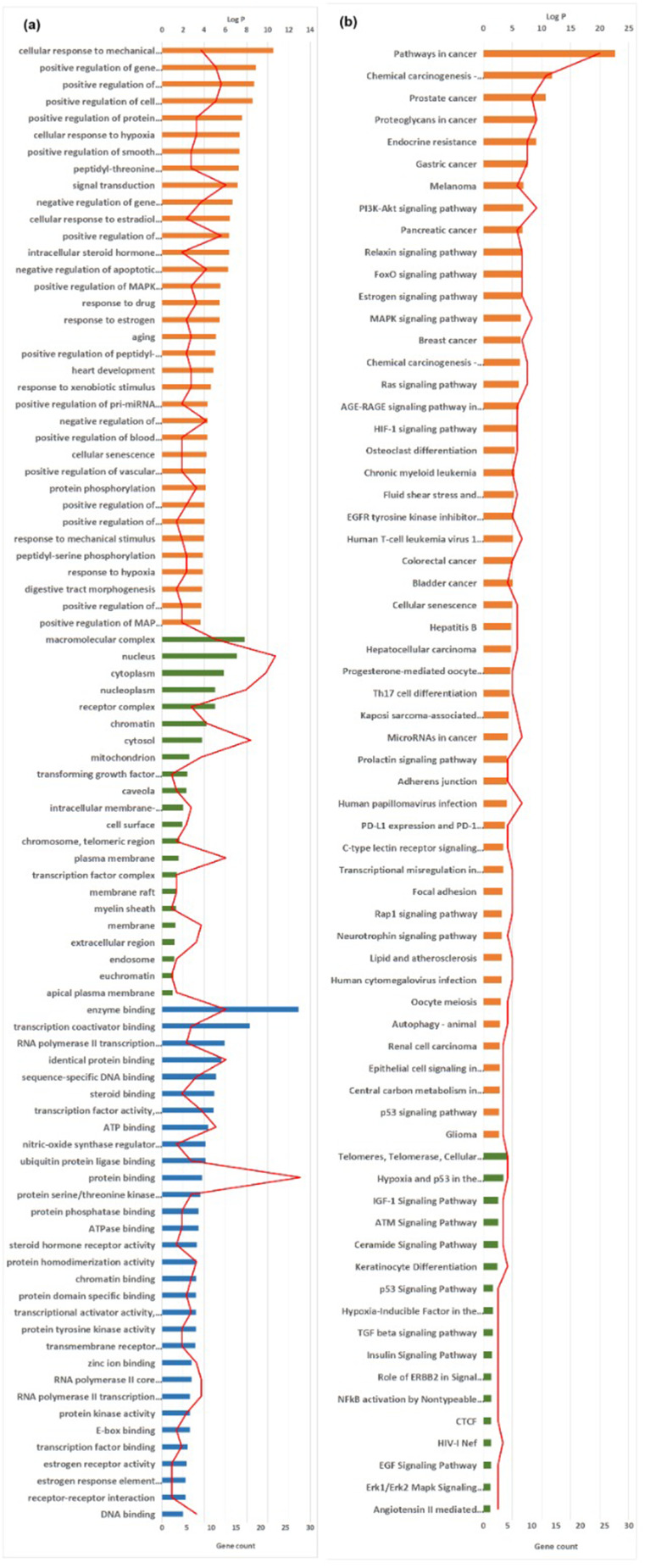
Enrichment analysis results of cancer-related targets in *Artemisia annua*. (**A**) BP (orange), CC (green) and MF (blue) results in GO annotation analysis. Y-axis stands for gene count (columns) and Log P values (red line), while the x-axis represents the genes in the GO annotation results. (**B**) Pathway enrichment bar chart where enriched pathways of the targets are represented by y-axis, and the x-axis stands for KEGG (orange) and BIOCARTA (green) pathways. Gene count is presented in columns and probabilities are denoted in a red line

To investigate the biological functions and pathway of the key targets of *Artemisia annua*, molecular functions (MF), biological process (BP), cellular components (CC) and KEGG were performed through the functional annotation tool of DAVID. As shown in Fig. (6a), 35 BPs were mainly regulated by cancer involving cellular response to mechanical stimulus, positive regulation of gene expression and positive regulation of transcription, DNA-templated. It was recently reported that a cytotoxic effect can be achieved when excessive levels of exogenous mechanically forced stimulation that used deep tissue-penetrating and focusable energy sources (e.g., ultrasound and magnetic fields) were directed to a cell [26]. Furthermore, regulation of the YAP and TAZ (YAP/TAZ) proteins controlled the cellular response to different mechanical stimuli causing its death [27]. Previous literature reviews confirmed the contribution of AML1, Sp1, and p300 in cancer through promoting E-cadherin expression in breast cancer cells thus, reducing cell motility [28]. Anqi Ge et al. conferred the possible antitumor activity of some intrinsic metabolites in herbal drugs such as baicalin by regulating DNA-templated RNA transcription in MCF-7 cancer cells which may afford new a therapeutic target in treating breast cancer [29]. Another study expressed the potential role of KIF1A in the positive regulation of DNA-templated RNA transcription via cell adhesion molecules (CAMs) and primary immunodeficiency. This mechanism suggested its contribution as one of the biomarkers causing poor prognosis in ovarian cancer [30].

Among the observed 22 CC terms, macromolecular complex, nucleus and cytoplasm were the most prominent cancer- related components. Enrichment results also revealed 31 molecular function items, including enzyme binding, transcription coactivator binding and RNA polymerase II transcription factor activity.

GO enrichment pathways analysis retrieved 50 pathways (Fig. 6b) from which, 17 BIOCARTA pathways were obtained where pathways in cancer, Chemical carcinogenesis, Prostate cancer and Proteoglycans in cancer were strikingly related to cancer.

### Molecular docking analysis of top-hit compounds in the active sites of the most enriched cancer-associated target genes

Prior to docking the top hit compounds into the most enriched target genes, a validation process was performed to determine the binding site and evaluate the ability of the docking program to reproduce the orientation of the native co-crystallized ligand. This was attained by extraction and re-docking of the co-crystallized ligands into their respective enzyme’s active site. Results revealed that the used docking software (Glide) was accurate in reproducing the native co-crystallized orientation of the redocked ligands with RMSD values ranging from 0.1 to 0.4 Å (Table 3, Figure S1).

**Table 3:** XP G scores of the top hit compounds in the compound-target network against the most enriched cancer-associated target proteins. RMSD values of the redocked co-crystallized ligands were also illustrated

	Nuclear factor NF-kappa-B (1LV2)	Mitogen-activated protein kinase 1 (4AN9)	Androgen receptor (2PIW)	P53-binding protein (4IPF)	Cyclin-dependent kinase 2 (1B39)	Cyclooxygenase-2 (3LN1)
4-Cadinene-3,11-diol; 3-(2-methylpropanoyl)	-5.607	-5.164	**-9.869**	**-4.836**	-6.324	**-8.683**
Artemisinin G	-4.213	-4.395	-8.360	-3.876	-3.524	-7.371
O-(2-Propenal) coniferaldehyde	-6.250	-6.630	-7.389	-3.308	-4.974	-6.174
O-(2-Glyceryl) coniferaldehyde	**-7.253**	**-8.676**	-8.796	-4.643	**-6.396**	-7.283
Arteamisinin III	-3.404	-3.670	-8.644	-3.534	-2.344	-6.300
Co-crystallized ligand	Palmitic acid	XL518 (GDC-0973)	Dihydrotestosterone	RO5045337	ATP	celecoxib
RMSD (A^o^)	0.341	0.146	0.162	0.237	0.183	0.247

The Glide module of the Schrodinger suite software was then used for calculating the docking XP G scores of *A. annua* hit compounds 4-cadinene-3,11-diol; (1α,3xi,6α,7β,10α)-form, 3-(2-methylpropanoyl), artemisinin G, O-(2-propenal) coniferaldehyde, (2-glyceryl)-O-coniferaldehyde and arteamisinin III against the active sites of the most enriched cancer-associated target genes NFKB1, MAP2K1, AR, MDM2, CDK2 and PTGS2. From Table 1, it can be observed that 4-cadinene-3,11-diol; 3-(2-methylpropanoyl) had the lowest XP G score against androgen receptor, P53-binding protein and cyclooxygenase-2. Whereas (2-glyceryl)-O-coniferaldehyde exhibited the most stabilized interaction with nuclear factor NF-kappa-B, mitogen-activated protein kinase and cyclin-dependent kinase 2.

**Fig. 7:**
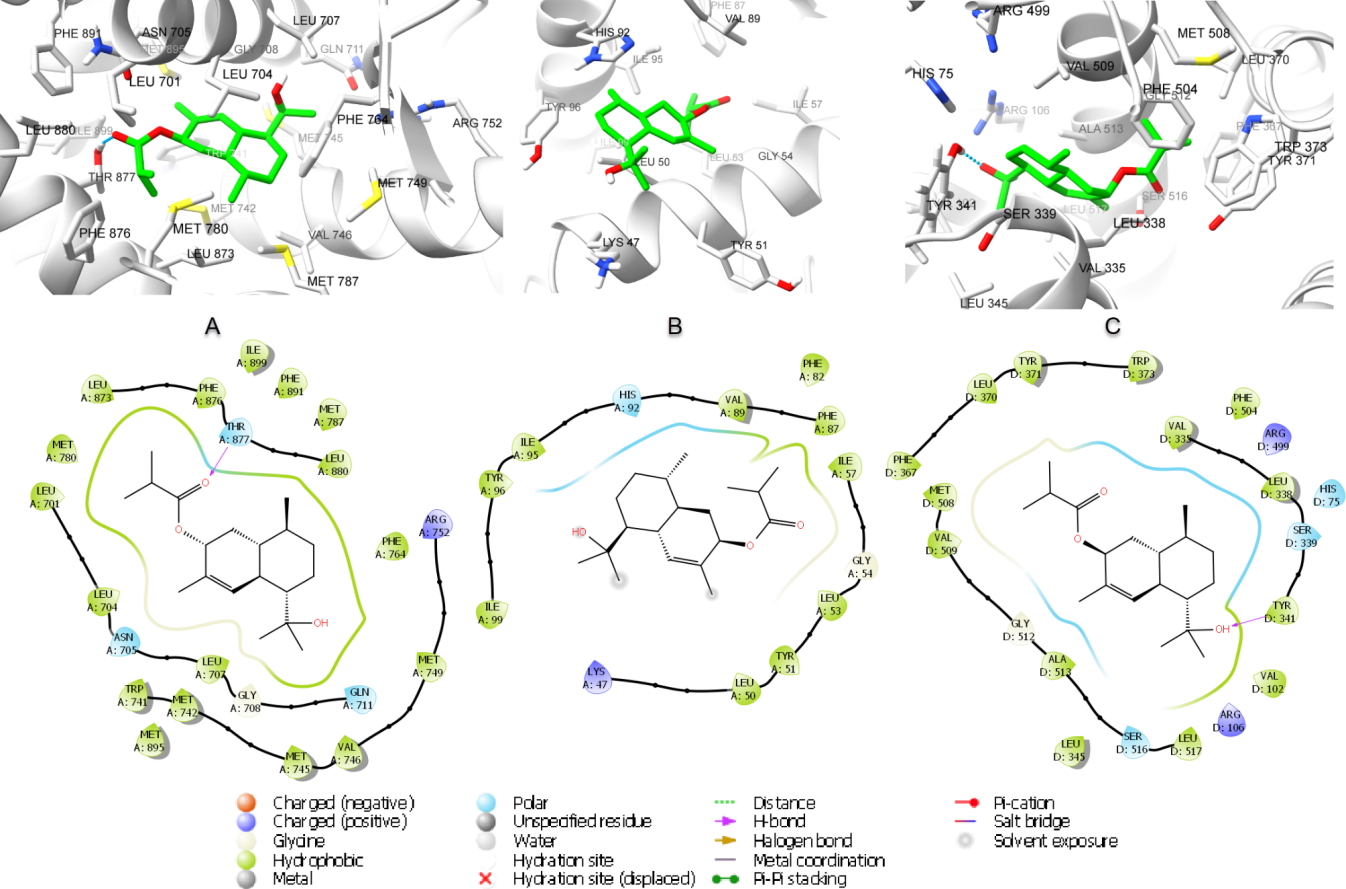
2D and 3D interaction diagrams of 4-Cadinene-3,11-diol; 3-(2-methylpropanoyl) in the active site of (**A**) androgen receptor (PBD ID 2PIW), (**B**) P53-binding protein (PDB ID 4IPF) and (**C**) cyclooxygenase-2 (PDB ID 3LN1).

The 2D and 3D interaction diagrams of 4-cadinene-3,11-diol; 3-(2-methylpropanoyl) in the active site of androgen receptor (PBD ID 2PIW) (Fig. 7A) revealed the formation of a hydrogen bond between ketone group and Thr877. Polar interactions were also observed with Gln711 and Asn705 in addition to hydrophobic interactions with Leu701, Leu707, Trp741, Met742, Val746, Met749, Met780, Met787 and Phe876.

Meanwhile, the interaction of 4-cadinene-3,11-diol; 3-(2-methylpropanoyl) with P53-binding protein (PDB ID 4IPF) involved charged positive interaction with Lys47. In addition to a polar interaction with His92. There were also hydrophobic interactions with Leu50, Tyr51, Leu53, Ile57, Val71, Phe87, Val89, Ile95, Tyr96 and Ile99 (Fig. 7B).

Furthermore, 4-cadinene-3,11-diol; 3-(2-methylpropanoyl) interacted cyclooxygenase-2 (PDB ID 3LN1) using one hydrogen bonds between ketonic group and Thr877. In addition to polar interactions with Asn705 and Gln711 and hydrophobic interaction with Leu701, Leu704, Leu707, Trp741, Met742, Val746, Phe764, Met780, Met787, Leu873, Phe876, Leu880 and Met895. There was also a charged positive interaction with Arg752 (Fig. 7C).

**Fig. 8:**
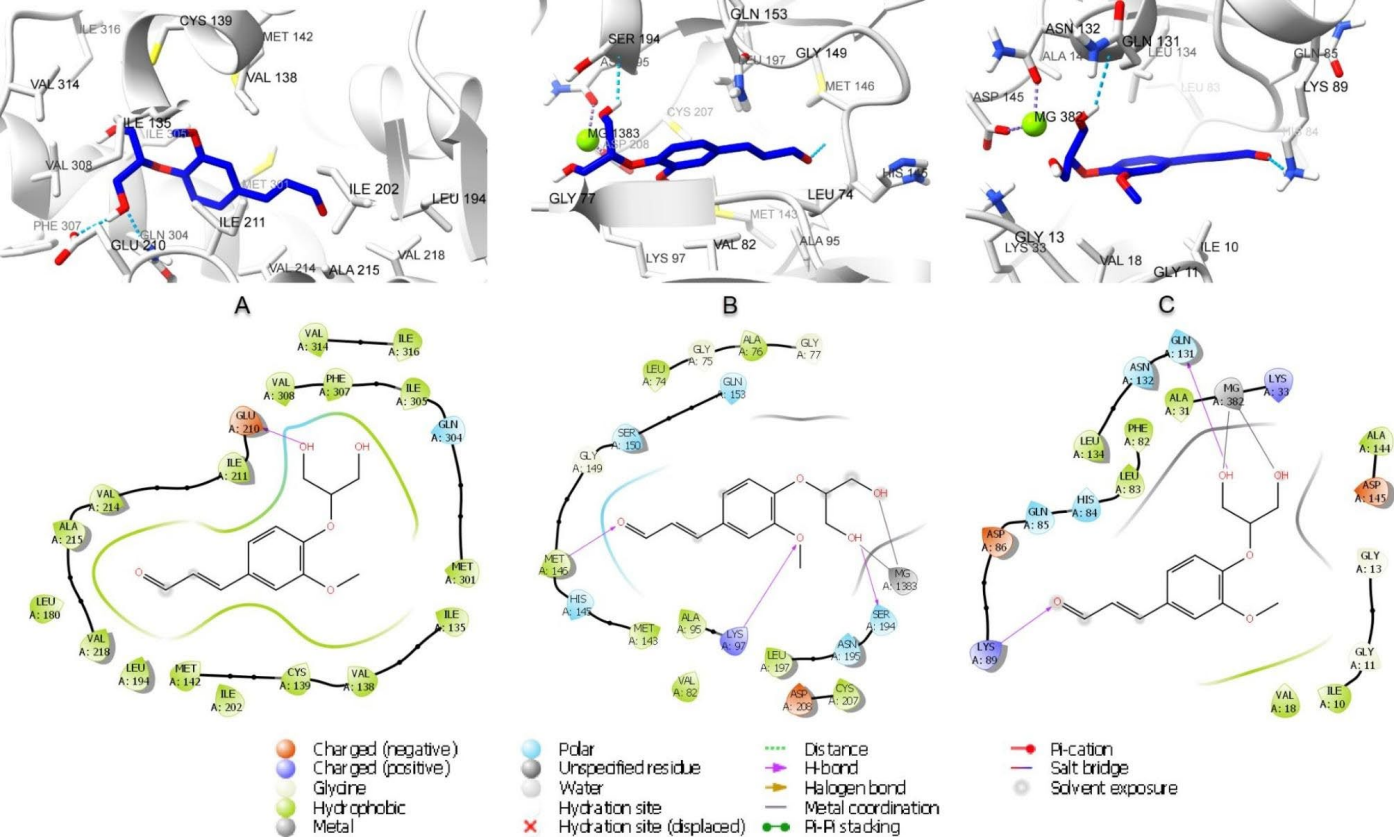
2D and 3D interaction diagrams of O-(2-glyceryl) coniferaldehyde in the active site of (**A**) nuclear factor NF-kappa-B (PDB ID 1LV2), (**B**) mitogen-activated protein kinase 1 (PDB ID 4AN9) and (**C**) Cyclin-dependent kinase 2 (PDB ID 1B39)

Whereas the stabilization of (2-glyceryl)-O-coniferaldehyde interaction with nuclear factor NF-kappa-B (PDB ID 1LV2) was through the formation of two hydrogen bonds between hydroxyl group in the glyceryl moiety and Glu210 and Gln304. There were also polar interactions with Gln304 and hydrophobic interactions with Ile135, Met142, Leu180, Leu194, Ile211, Val214, Ala215, Val218, Met301, Ile305, Phe307, Val308, Val314 and Val316. In addition to negatively charged interaction with Glu210 (Fig. 8A).

On the other hand, the interaction of (2-glyceryl)-O-coniferaldehyde with mitogen-activated protein kinase 1 (PDB ID 4AN9) involved three hydrogen bonds between aldehydic ketone and Met146 residue, hydroxyl group in the glyceryl moiety and Ser194 and between methoxyl oxygen and Lys97. There were also hydrophobic interactions with Leu74, Ala76, Val82, Ala95, Met143, Met146, Leu197 and Cys207 residues and polar interaction with Asn78, Hie145, Ser150, Asn195 and Ser194. In addition to a charged negative interaction with Glu144 and Asp208 and charged positive ones with Lys97 and Lys 192(Fig. 8B). While the interaction of (2-glyceryl)-O-coniferaldehyde with cyclin-dependent kinase 2 (PDB ID 1B39) was stabilized via the formation of a hydrogen bond between aldehydic ketone group and Lys89 and between hydroxyl group in the glyceryl moiety and Gln131. In addition to the formation of coordination reaction with Mg382 and hydrophobic interactions with Ile10, Val18, Ala31, Phe82, Leu83, Leu134 and Ala144. There were also a charged positive interaction with Lys 33 and Lys89 and negative ones with Glu12, Asp86 and Asp145. Moreover, polar interactions with Hie84, Gln85, Gln131 and Asn132 were also observed (Fig. 8C).

### Molecular dynamics simulations stability analyses of hit protein-ligand complexes

**Fig. 9:**
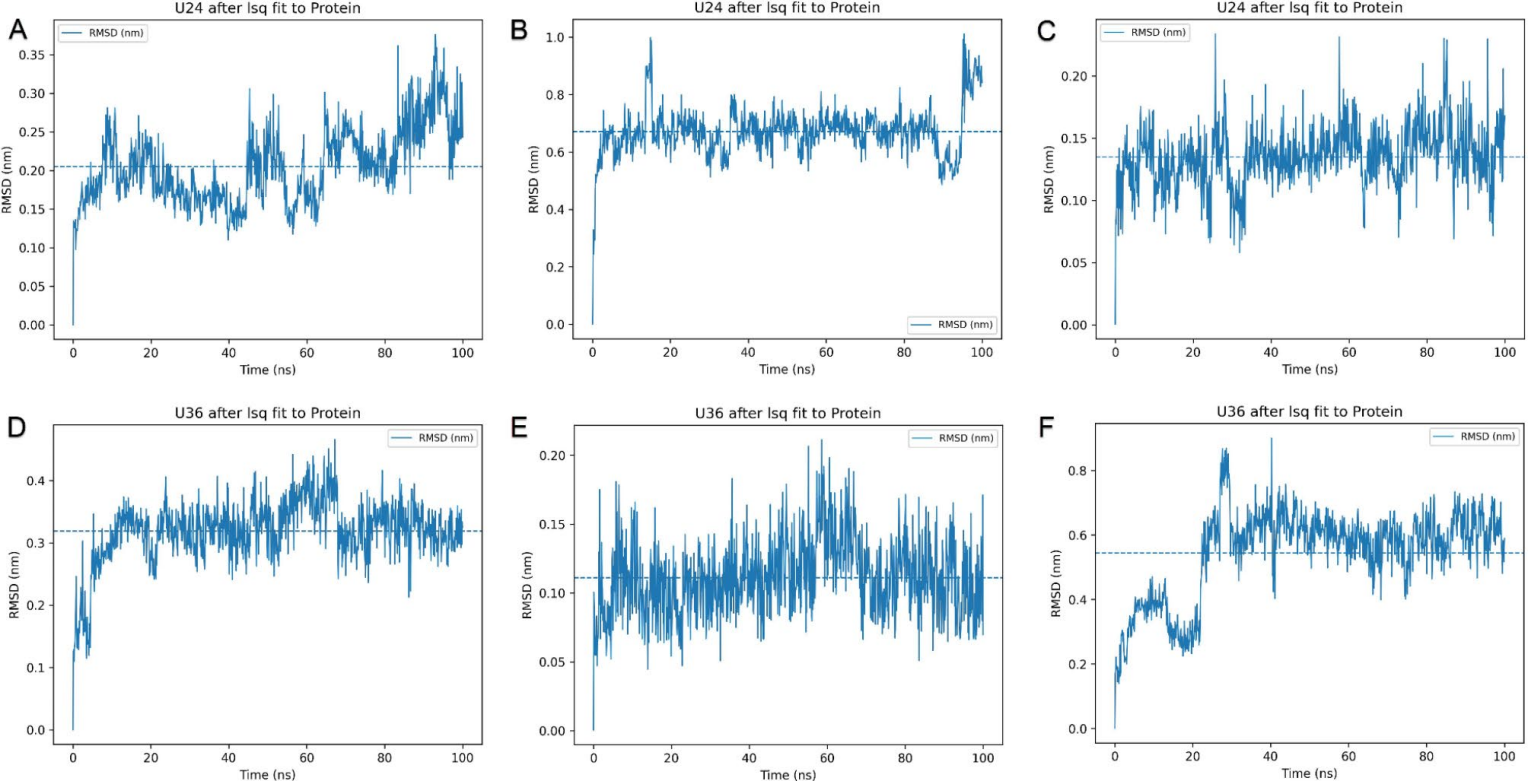
RMSD analysis numerically showing the stability of compounds 4-Cadinene-3,11-diol; 3-(2-methylpropanoyl) and O-(2-glyceryl) coniferaldehyde relative to proteins from molecular dynamics’ simulations. (**A**-**B**-**C**) RMSD of 4-Cadinene-3,11-diol; 3-(2-methylpropanoyl) with androgen receptor, P53-binding and cyclooxygenase-2. (**D**-**E-F**) RMSD of O-(2-glyceryl)-coniferaldehyde with nuclear factor NF-kappa-B, mitogen-activated protein kinase 1 and cyclin-dependent kinase 2 for 100 ns, respectively

Molecular dynamics simulations are a molecular modeling technique often used to in silico [31, 32] explain the stability of protein-ligand complexes [33]. In this study, the compound 4-cadinene-3,11-diol; 3-(2-methylpropanoyl) with target proteins androgen receptor, P53-binding and cyclooxygenase-2, and target proteins of compound (2-glyceryl)-O-coniferaldehyde with nuclear factor NF-kappa-B, mitogen-activated protein kinase 1 and cyclin-dependent kinase 2 protein-ligand complexes created by molecular docking were analyzed by molecular dynamics simulation of 100 ns duration. RMSD measurements were used to quantify the changes and movements of compounds in protein active pockets per frame [34]. The RMSD value between 4-cadinene-3,11-diol; 3-(2-methylpropanoyl) and the androgen receptor was below 0.3 nm up to 80 ns and averaged 0.21 as shown in (Fig. 9A). 4-Cadinene-3,11-diol; 3-(2-methylpropanoyl) and P53-binding complex RMSD peaked below 0.7 nm up to 90 ns and then at 1 nm as shown in (Fig. 9B) and averaged 0.67. As given in (Fig. 9C), 4-Cadinene-3,11-diol; 3-(2-methylpropanoyl) at the cyclooxygenase-2 active site generally gave a value below 0.2 nm, with an average RMSD of 0.13 nm. The RMSD of the second compound, (2-glyceryl)-O-coniferaldehyde, was measured below 0.4 nm with a mean of 0.32, as shown in (Fig. 9D), according to the nuclear factor NF-kappa-B. (2-glyceryl)-O-coniferaldehyde and mitogen-activated protein kinase 1 gave RMSD value below 0.2 nm and an average of 0.11 nm as shown in (Fig. 9E). With the other target protein cyclin-dependent kinase 2, the first 25 ns fluctuated up to 0.4 nm, then elevated with mean RMSD value of 0.54 as shown in (Fig. 9F).

**Fig. 10:**
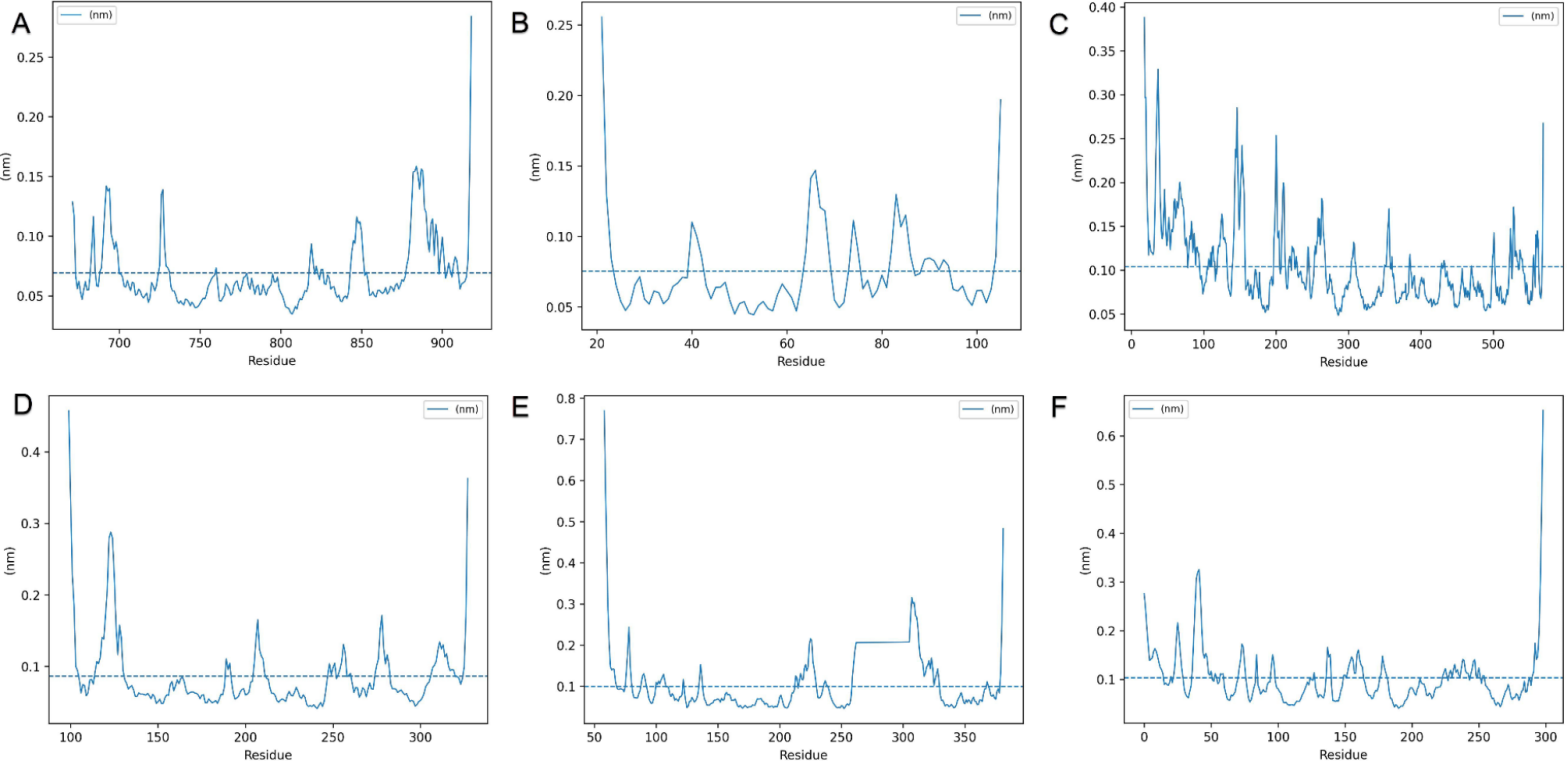
RMS fluctuation analyzes for flexibility and mobility in protein structures. (**A**-**B**-**C**) RMSF of 4-Cadinene-3,11-diol; 3-(2-methylpropanoyl) with androgen receptor, P53-binding and cyclooxygenase-2. (**D**-**E-F**) RMSF of O-(2-glyceryl)-coniferaldehyde with nuclear factor NF-kappa-B, mitogen-activated protein kinase 1 and cyclin-dependent kinase 2 for 100 ns, respectively

RMSF analysis, which calculates the mean square deviation of each amino acid residue from its initial position, provides an indication of the degree of flexibility or rigidity in distinct regions of a protein. This methodology was utilized to ascertain the average flexibility of the target proteins in this study. In the initial phase of the investigation, the interaction between the compound 4-Cadinene-3,11-diol; 3-(2-methylpropanoyl) and the target proteins, namely the androgen receptor, P53-binding protein, and cyclooxygenase-2, was analyzed (Fig. 10A-B-C). The data indicated that the average RMSF value for cyclooxygenase-2 was 0.104 nm, which was notably higher than that of the other target proteins. This suggests that cyclooxygenase-2 displayed a higher degree of average flexibility in comparison to the androgen receptor and P53-binding protein. The average RMSF values for the androgen receptor and P53-binding protein were computed to be 0.069 and 0.075 nm, respectively. These findings denote that these proteins showed a comparable degree of average flexibility, with a marginally higher value noted in the P53-binding protein. In the subsequent phase of the investigation, the interactions between the compound O-(2-glyceryl) coniferaldehyde and a different set of target proteins, namely nuclear factor NF-kappa-B, mitogen-activated protein kinase 1, and cyclin-dependent kinase 2, were explored (Fig. 10D-E-F). It was found that the average RMSF value for cyclin-dependent kinase 2, at 0.103 nm, was the highest among these target proteins, suggesting that cyclin-dependent kinase 2 exhibited a greater degree of average flexibility compared to nuclear factor NF-kappa-B and mitogen-activated protein kinase 1. The average RMSF values for nuclear factor NF-kappa-B and mitogen-activated protein kinase 1 were computed to be 0.086 and 0.100 nm, respectively. This implies that these proteins showed similar degrees of average flexibility, albeit with a slightly higher average flexibility discerned in the mitogen-activated protein kinase 1.

**Fig. 11:**
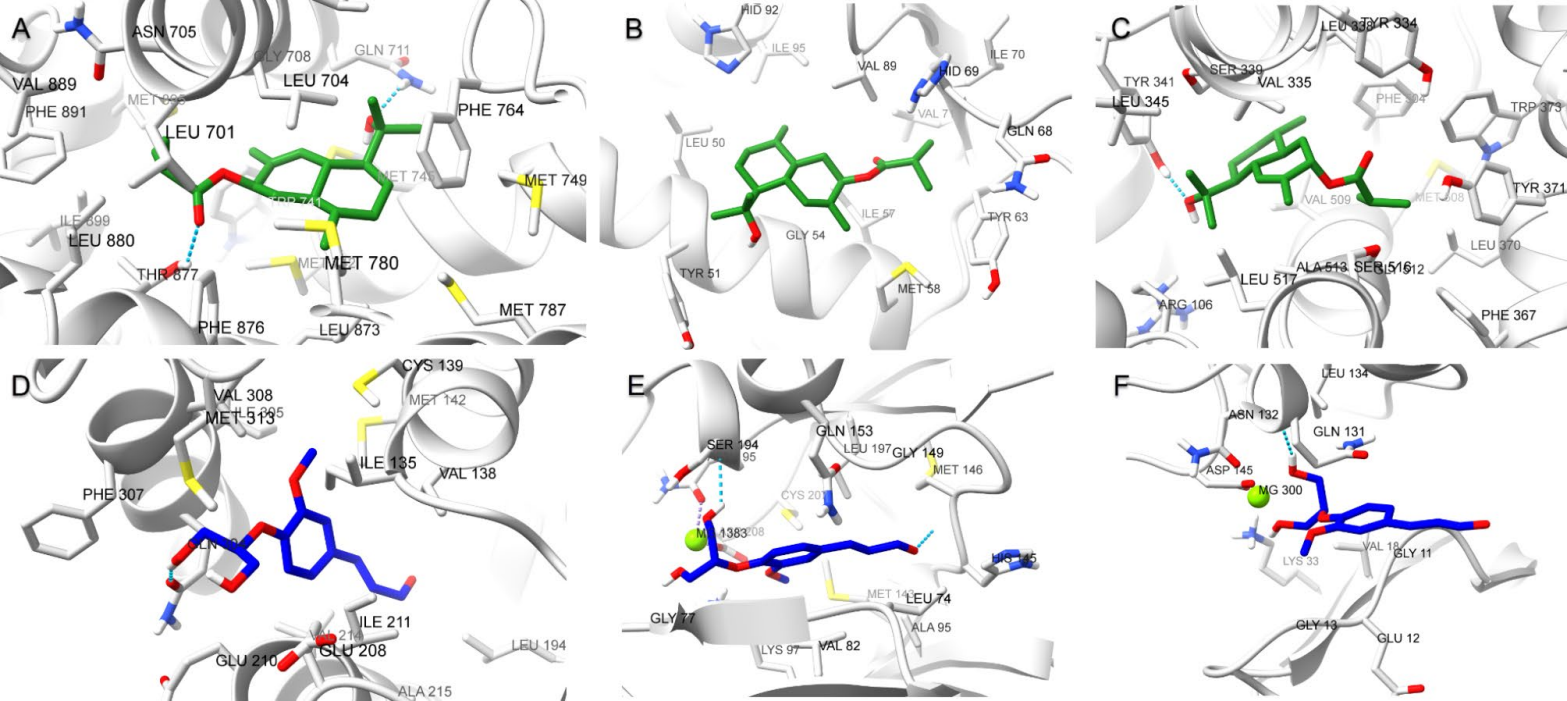
Protein-ligan interactions at the end of 100 ns molecular dynamics simulation. (**A**-**B**-**C**) Androgen receptor, P53-binding and cyclooxygenase-2 with 4-cadinene-3,11-diol; 3-(2-methylpropanoyl) and (**D**-**E**-**F**) Nuclear factor NF-kappa-B, mitogen-activated protein kinase 1, and binding poses of cyclin-dependent kinase 2 with (2-glyceryl)-O-coniferaldehyde, respectively

The binding poses of the protein-ligand complexes at the end of 100 ns were analyzed to examine interaction changes [35]. As shown in (Fig. 11A), 4-cadinene-3,11-diol; 3-(2-methylpropanoyl) and the androgen receptor molecular docking pose was stabilized by hydrogen bonding with Thr877(4.05 Å), and Gln711(4.15 Å) and Met745 (3.47 Å), and continued hydrophobic interactions with Leu701, Asn705, Gly708, Trp741, Met742, Val746, Met749, Met780, Met787 and Phe876. Menwhile, 4-cadinene-3,11-diol;3-(2-methylpropanoyl) and P53-binding protein complex, as shown in (Fig. 11B), was stabilized by hydrophobic interactions with Leu50, Thr51, Met58, Gly54, Ile57, Leu62, Thr63, Gln68, His69, Ile70, Val71, Val89, His92 and Ile95. The hydroogen bond between the hydroxyl group of 4-cadinene-3,11-diol; 3-(2-methylpropanoyl) and Thr341 of cyclooxygenase-2 remained stable, and hydrophobic interactions with Val102, Ser339, Phe367, Met508 and Ser516 has been preserved as shown in (Fig. 11C).

Regarding the other compound (2-glyceryl)-O-coniferaldehyde and the target nuclear factor NF-kappa-B, the hydrogen bond with Glu210 and Gln304 remained stable at the 100th ns, as shown in (Fig. 11D), and hydrophobic interactions with Ala215, Met301, Ile305, Phe307, Val308 continued. Metal-acceptor interaction between (2-glyceryl)-O-coniferaldehyde and mitogen-activated protein kinase 1 with Mg^++^ (2.12 Å and 2.10 Å), hydrogen bond with Ser194 (3.97 Å) and Met146 (4.24 Å) interactions are preserved as shown in (Fig. 11E). As given (Fig. 11F), the metal-acceptor interaction with Mg^++^ (1.99 Å and 2.13 Å) and the hydrogen bond interaction with Gln131 remained stable between the other target cyclin-dependent kinase 2 and (2-glyceryl)-O-coniferaldehyde, as in the molecular docking pose. Finally, to examine the stability states of these 6 protein-ligand complexes, molecular dynamic animation videos were created from 250 frames recorded for 100 ns and are given in Supporting Information (Video S1-6) [36]. According to post-molecular dynamics simulation trajectory analyses, the compounds 4-cadinene-3,11-diol; 3-(2-methylpropanoyl) and (2-glyceryl)-O-coniferaldehyde have the potential to form stable interactions with selected target proteins.

### Assessment of the in-vitro cytotoxicity activity of ***A.annua*** extract

#### Cytotoxicity of ***A.annua*** on healthy (Vero E6) cells

**Fig. 12:**
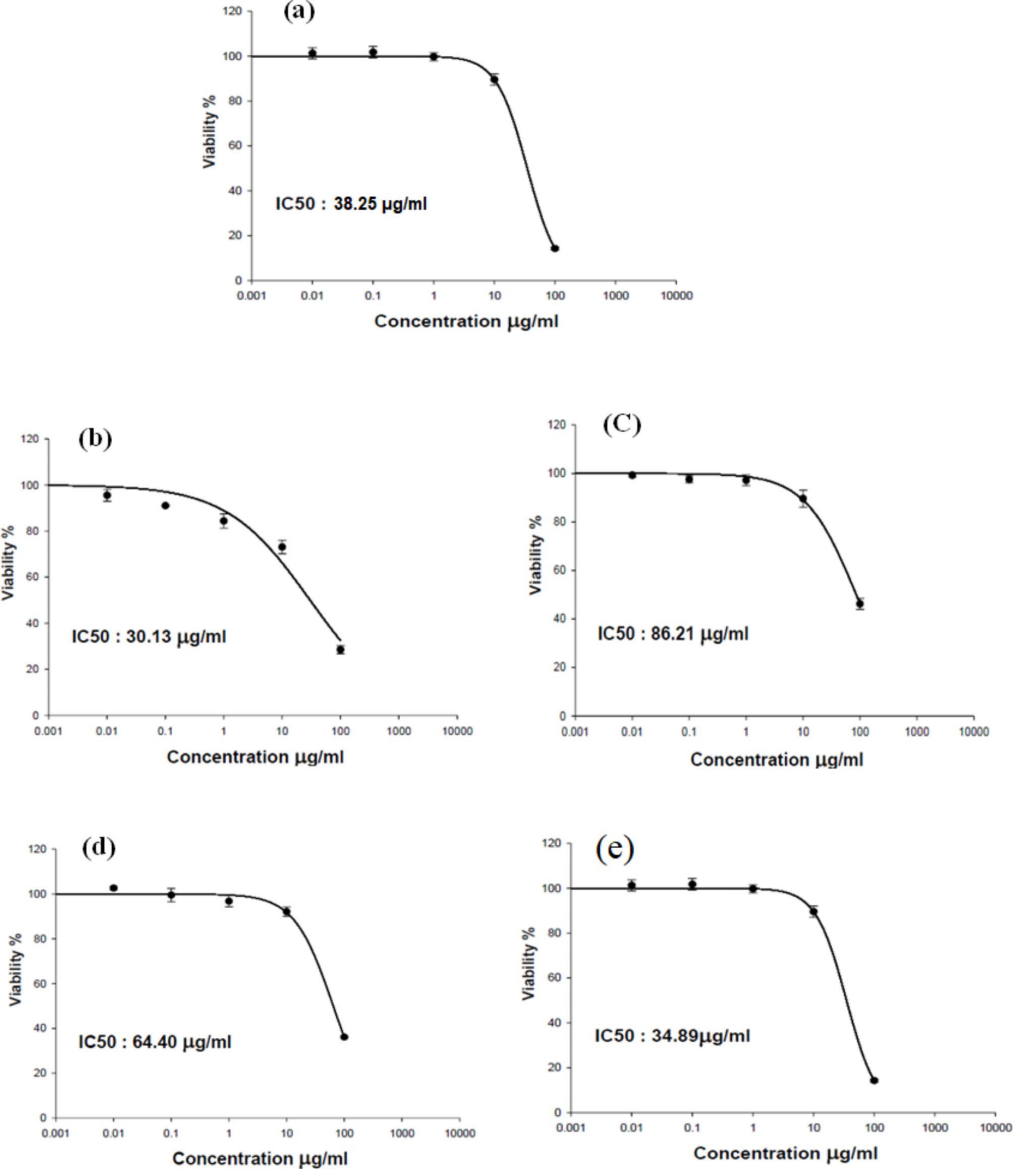
Dose response curve of the cytotoxic activity of *A.annua* extract on healthy cells (Vero E6) (**a**), human prostate (PC-3) (**b**), breast (MDA-MB-231) (**c**), pancreatic (PANC-1) (**d**) and melanoma (A375) (**e**) cell lines

The effect of *A.annua* extract on the viability of Vero E6 cells was evaluated using MTT assay. It showed 50% cytotoxicity (CC_50_) at concentration 38.25 μg/mL (i.e. more than 30 μg/mL) indicating their safety on these cells [37] (Fig. 12a).

#### In vitro anticancer activity of ***A.annua*** extract on human prostate (PC-3), breast (MDA-MB-231), pancreatic (PANC-1) and melanoma (A375) cancer cell lines

Artemisia extract was subjected to in vitro anticancer activity testing on human prostate (PC-3), breast (MDA-MB-231), pancreatic (PANC-1) and melanoma (A375) cancer cell lines. The results shown in (Fig. 12b-e) indicated variable potency of artemisia against the studied cancer types, where it showed high potency against human prostate (PC-3) cell lines with an IC_50_ value of 30.13 μg/mL, followed by breast (MDA-MB-231), pancreatic (PANC-1) and melanoma (A375) cancer cell lines scoring IC_50_ values 86.21, 64.40 and 34.89 μg/mL respectively. These values were moderately competing to those of doxorubicin (IC_50_ values 6.4, 4.1, 43 and 0.5 μg/mL, respectively). Results also showed concordance with the literature survey where it was suggested that artesunate – a semi-synthetic artemisinin derivative - suppressed tumor growth of prostatic cancer cells (PC-3) via modulating AR-DNMT3b pathway [38]. In the same context, artemisinin exhibited anti-proliferative effects in prostate cancer cells through transcriptional down-regulation of CDK4 expression by disruption of Sp1 interactions with the CDK4 promoter [39]. Other study confirmed that *Artemisia annua* extract exerted a concentration-dependent effect on breast (MCF-7) cancer cells [40], however, its isolated flavonol Chrysosplenol d activated ERK1/2, and induced autophagy in MDA-MB-231 (breast cancer) cells [41]. The total polyphenolic fraction inhibited the proliferation of the same cell line either through suppression of vascular cell adhesion molecule-1 [42] or stem Cell Phenotype, β-Catenin, and MMP-9 [6]. Significant inhibition of melanoma and breast cancer cells by some other constituents including, artemisinin, scopoletin, 1,8-Cineole, arteanuine B, artemisitene was also reported [43].

KEGG pathway analysis of human prostate (ID: hsa05215) (Figure S2), breast (ID: hsa05224) (Figure S3), melanoma (ID: hsa05212) (Figure S4) and pancreatic cancer (ID: hsa05218) (Figure S5) illustrated the potential targets and pathways of *A. annua* chemical constituents where the orange ovals indicated the targets where the molecules interacted and the red rectangles indicated the targeted pathways [44–46].

## Materials and methods

### Network pharmacology analysis

#### Collection of ***Artemisia annua*** compounds and corresponding targets

Dictionary of natural products database (http://dnp.chemnetbase.com/, CRC press) is a platform containing many compounds from herbal medicine [47]. It was used to collect the potential active eighty components of *Artemisia annua* (table S3). Protein targets interacting with those potential active compounds were anticipated by importing the molecular structure files into Swiss Target Prediction (http://www.swisstargetprediction.ch/) databases. Probability score was used to evaluate the interaction strength of the compound to each target.

#### Sorting cancer-related target genes

By searching for “cancer” in the GeneCards database (https://www.genecards.org), targets related to cancer were identified and a disease-target database was constructed.

#### Screening common drug and disease targets

The online Venny 2.1.0 program (https://bioinfogp.cnb.csic.es/tools/venny/) was employed to identify intersecting Artemisia annua and cancer targets, through which, Venn diagrams were drafted.

#### Pathway enrichment analysis and protein-protein interaction (PPI) network

Kyoto Encyclopedia of Genes and Genomes (KEGG) and protein-protein interaction (PPI) data were compiled and retrieved from String (https://string-db.org/), using the species “homo sapiens”. A minimum required interaction score to the highest confidence level (0.900) was set aiming to generate protein-protein interaction information.

#### Construction of the component-target-pathway network

Two networks namely, compound-gene-network and Gene-pathway-network were constructed via Cytoscape 3.7.2 software, displaying the active components, target genes, and pathways as nodes and the inter-node relationships as edges.

### KEGG pathway and gene ontology (GO) enrichment analysis

GO enrichment analysis is an important computational tool used to provide a logical framework of gene function [48]. It can effectively recognize the biological processes, cellular components and molecular functions related to a certain ailment and obtain more profound information about a list of genes [49]. KEGG pathway analysis and GO Enrichment analysis was conducted using functional annotation tool in Annotation, Visualization, and Integrated Discovery (DAVID) (https://david.ncifcrf.gov/) where ‘’ homo sapiens’’ species were selected. Prior to enrichment, genes names were converted into corresponding UniProt accession numbers. GO terms possessing p-values < 0.05 were considered statistically significant.

### Molecular docking analysis

The Protein Data Bank (PDB) was used for retrieving the crystal structure of the most enriched six targets revealed from network pharmacology analysis, which are Nuclear factor NF-kappa-B p105 subunit (1LV2), Mitogen-activated protein kinase 1 (4AN9), Androgen receptor (2PIW), P53-binding protein (4IPF), Cyclin-dependent kinase 2 (1B39) and Cyclooxygenase-2 (3LN1). The selection of crystal structure of each protein was relied on best resolution available. The 3D structures of top-five compounds reveled from network pharmacology were imported as SDF into the LigPrep module of Maestro 10.2 molecular modeling (Schrodinger®) software package to obtain low energy structures of compounds. Ionization at pH 7 was performed to produce all possible states. High-resolution crystal structures of protein targets were retrieved from Protein Data Bank (PDB). Protein preparation was accomplished using protein preparation module of Maestro where they were preprocessed by assigning bond orders and hydrogens in addition to removing all the water molecules beyond 5 °A from the active site. Assignment of H-bonds was performed via PROPKA at PH = 7, then energy minimization using OPLS 3 force field was performed till the relative mean standard deviation (RMSD) of the minimized structure compared to the crystal structure was above 0.30 °A [50]. Receptor grid generation module using boxes enclosing the centroids of co-crystallized ligands were set as the grids. Molecular docking analysis was achieved using Glide docking program of Maestro molecular modeling package implementing extra-precision (XP-Glide) mode. The binding modes of the compounds with targets were visualized using Maestro interface.

### Molecular dynamics simulations

The stability of the hit protein-ligand complexes was tested by molecular dynamics simulations performed with Gromacs v2021.2 [51]. System preparations such as solvation and neutralization of protein-ligand complexes obtained by molecular docking for molecular dynamics simulation and creation of topology files were done with the Solution Builder tool in the CHARMM-GUI server. Amber FF99SB [52, 53] was preferred for topology files. A temperature of 300 K and a pressure of 1 atm was equilibrated by the Nose-Hoover thermostat and Parrinello-Rahman barostat methods for 0.3 ns, and 100 ns molecular dynamics simulation was performed to 2 fs under PBC. Post molecular dynamic trajectory analysis with root mean square deviation (RMSD) and root mean square fluctuation (RMSF) gmx rms script, analysis and visualization of protein-ligand binding poses at 100 ns with [54] UCSF ChimeraX v1.4, and creation of animation videos with PyMOL Molecular Graphics System v2.4.1 were performed.

### In vitro anticancer activity screening of ***A. annua***

#### Preparation of ***A. annua*** extract

Aerial parts of *Artemisia annua* (1.5 kg) were collected from a cultivated area in Cairo. The plant material was identified by Mrs. Teresa Labib, Taxonomist at the El-Orman Botanical Garden, Giza. Our study complies with relevant institutional, national, and international guidelines and legislation. A voucher sample was kept at the Pharmacognosy Department, Faculty of pharmacy, Alexandria University. The plant was carefully air-dried to obtain 500 g that were extracted with 70% ethanol (10 g/100 ml) twice at room temperature (27.5^0^ C) by sonication for two hours. The extract was filtered and concentrated to dryness using the rotary evaporator at 40^o^C under vacuum then kept at 4^o^C before use.

#### Cytotoxicity of ***A. annua*** extract on healthy (Vero E6) cells

The dilution of the tested extract was done with Dulbecco’s Modified Eagle’s Medium (DMEM). The stock solutions of the tested extract were prepared in 10% DMSO in double distilled H_2_O. MTT method with minor modifications was used to evaluate the cytotoxic effects in Vero E6 cells (Green monkeys kidney cells) [55, 56]. In 96 well plates, Vero E6 cells were seeded (100 μl/well at a density of 3 × 10^5^ cells/ml) followed by their incubation for 24 h at 37 ^o^C in 5% CO_2_. After 24 h, treatment of cells with various concentrations of the tested extract in triplicates was done followed by their incubation for another 24 h. After that, the supernatant layer was discarded, and washing of cell monolayers with sterile PBS 3 times was carried out. To each well, MTT solution (20 μl of 5 mg/ml stock solution) was added followed by incubation at 37 °C for 4 h then medium aspiration. The formed formazan crystals were dissolved with 200 μl of acidified isopropanol (0.04 M HCl in absolute isopropanol, 0.073 ml HCl in 50 ml isopropanol). Finally, a multi-well plate reader was used to measure the absorbance of formazan solutions at λ_max_ 540 nm using 620 nm as a reference wavelength. The percentage of cell cytotoxicity 50 compared to the untreated cells was calculated with the following equation:

$${\rm{\% }}\,{\rm{CC50}}\,{\rm{ = }}\,\left( {\left( {{{\rm{A}}_{\rm{0}}}{\rm{ - A}}} \right){\rm{/}}{{\rm{A}}_{\rm{0}}}} \right)\,{\rm{*}}\,{\rm{100}}$$

A_0_ is the absorbance of cells without treatment.

A is the absorbance of cells with treatment.

The concentration which exhibited 50% cytotoxicity (CC50) was determined by plotting % cytotoxicity versus sample concentration.

#### In vitro sulforhodamine B (SRB) cytotoxicity assay

Human prostate (PC-3), breast (MDA-MB-231), pancreatic (PANC-1) and melanoma (A375) cancer cells were separately preserved in Dulbecco’s Modified Eagle Media (DMEM) supplied with 100 units per mL of penicillin, 100 mg/mL of streptomycin, and 10% of heat-inactivated fetal bovine serum in humidified, 5% (v/v) CO2 atmosphere at 37 °C. Sulforhodamine B (SRB) assay was applied for assessment of cell viability [57, 58] Aliquots of 100 mL cell suspension (5 × 10^3^ cells) from each cell line were separately placed in 96-well plates and incubated for 24 h in complete media. Then treatment of the cells with another aliquot of 100 mL media containing *A.annua* at 0.001, 0.01, 0.1, 1, 10, 100 mg/mL concentrations was carried out. After 72 h exposure to drug, cells were fixed by exchanging media with 150 mL of 10% trichloroacetic acid (TCA) and then the cells were incubated at 4 °C for 1 h. The TCA solution was removed, and distilled water was used for washing the cells 5 times. Aliquots of 70 mL SRB solution (0.4% w/v) were added and incubated in a dark place at room temperature for 10 min. Washing the plates 3 times with 1% acetic acid was carried out, then the plates were allowed to air-dry overnight. After that, protein-bound SRB stain was dissolved using 150 mL of TRIS (10 mM); finally, the measurement of the absorbance was performed at 540 nm by the use of a BMG LABTECH^®^ –FLUO star Omega microplate reader (Ortenberg, Germany). The whole experiment has been conducted in triplicate for each tested concentration to attain reproducibility. Doxorubicin was used as a reference anti-cancer drug.

## Conclusion

Owing to the multi-chemical components of the plant extracts, their multi-pharmacological effects as well as their multi-action targets in the treatment of different diseases, network pharmacology had combined bioinformatics and pharmacology organically to clarify and explain the multi-mechanisms of action of these plants via emphasizing the relationship between targets, components and diseases. In this study, the network pharmacology-based analysis of *A. annua* showed that the hit phytoconstituents related to cancer targets were 3-(2-methylpropanoyl)-4-cadinene-3,11-diol, artemisinin G, O-(2-propenal) coniferaldehyde, (2-glyceryl)-O-coniferaldehyde and arteamisinin III, whereas the main cancer allied targets were NFKB1, MAP2K1, AR, MDM2, CDK2 and PTGS2. Sixty-eight significant signaling KEGG pathways were recognized where the most enriched ones were prostate cancer, breast cancer, melanoma and pancreatic cancer. Among hit compounds, molecular docking and dynamic simulation studies revealed that 4-cadinene-3,11-diol; 3-(2-methylpropanoyl) had the lowest XP G score against androgen receptor, P53-binding protein and cyclooxygenase-2. Whereas (2-glyceryl)-O-coniferaldehyde exhibited the most stabilized interaction with nuclear factor NF-kappa-B, mitogen-activated protein kinase and cyclin-dependent kinase 2. This study represents a thorough explanation of the proposed mechanism of action of *A. annua* phytoconstituents in cancer and suggests this natural product as a potential source for cancer prevention or treatment. making it worthy of clinical application and promotion. However, only part of the specific mechanism of action has been clinically verified and further extensive in vivo and clinical studies are required to confirm the anti-cancer potential of the concluded top hit natural compounds.

### Electronic supplementary material

Below is the link to the electronic supplementary material.

**Supplementary Material 1: Figure S1**: 3D interaction diagrams between the top six target protein crystal structures with top hit ligands together with the co-crystallized ligands for molecular docking validation.

**Supplementary Material 2: Figure S2**: KEGG pathway analysis of human prostate cancer (ID: hsa05215) illustrating the potential targets and pathways of A. annua chemical constituents where the orange ovals indicated the targets where the molecules interacted and the red rectangles indicated the targeted pathways.

**Supplementary Material 3: Figure S3**: KEGG pathway analysis of human breast cancer (ID: hsa05224) illustrating the potential targets and pathways of A. annua chemical constituents where the orange ovals indicated the targets where the molecules interacted and the red rectangles indicated the targeted pathways.

**Supplementary Material 4: Figure S4**: KEGG pathway analysis of human melanoma (ID: hsa05212) illustrating the potential targets and pathways of A. annua chemical constituents where the orange ovals indicated the targets where the molecules interacted and the red rectangles indicated the targeted pathways.

**Supplementary Material 5: Figure S5**: KEGG pathway analysis of human pancreatic cancer (ID: hsa05218) illustrating the potential targets and pathways of A. annua chemical constituents where the orange ovals indicated the targets where the molecules interacted and the red rectangles indicated the targeted pathways.

**Supplementary Material 6: Video S1**: MD Simulation animation of 250 snapshots between 0 and 100 ns of androgen receptor with 4-cadinene-3,11-diol; 3-(2-methylpropanoyl) (PDB ID: 2PIW). **Video S2**. MD Simulation animation of 250 snapshots between 0 and 100 ns of P53-binding protein with 4-cadinene-3,11-diol; 3-(2-methylpropanoyl) (PDB ID: 4IPF). **Video S3**. MD Simulation animation of 250 snapshots between 0 and 100 ns of cyclooxygenase-2 with 4-cadinene-3,11-diol; 3-(2-methylpropanoyl) (PDB ID: 3NL1). **Video S4**. MD Simulation animation of 250 snapshots between 0 and 100 ns of NF-kappa-B with O-(2-Glyceryl)-coniferaldehyde (PDB ID: 1LV2). **Video S5**. MD Simulation animation of 250 snapshots between 0 and 100 ns of mitogen-activated protein kinase 1 with O-(2-Glyceryl)-coniferaldehyde (PDB ID: 4AN9). **Video S6**. MD Simulation animation of 250 snapshots between 0 and 100 ns of cyclin-dependent kinase 2 with O-(2-Glyceryl)-coniferaldehyde (PDB ID: 1B39).

**Supplementary Material 7: Table S1**: Cancer-related target genes retrieved from Genecards database.

**Supplementary Material 8: Table S2**: Protein-Protein interactions retrieved from String database.

**Supplementary Material 9: Table S3**: Database of A. annua active constituents and their SMILES.

## Data Availability

All data generated or analyzed during this study are included in this article (and its supplementary information files).
