# Supplementary figures and images for: Computational biology and in vitro studies for anticipating cancer-related molecular targets of sweet wormwood (Artemisia annua)
Source: BMC Complement Med Ther. 2023 Sep 8;23:312. doi: 10.1186/s12906-023-04135-0 (PMC10492370; doi:10.1186/s12906-023-04135-0)

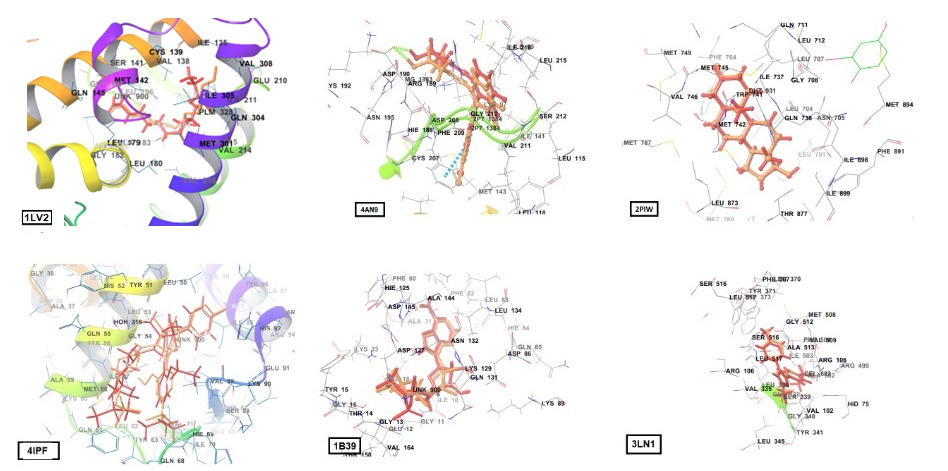

Supplement: Supplementary file 1 — Supplementary Material 1: Figure S1: 3D interaction diagrams between the top six target protein crystal structures with top hit ligands together with the co-crystallized ligands for molecular docking validation. [file 12906_2023_4135_MOESM1_ESM.docx]

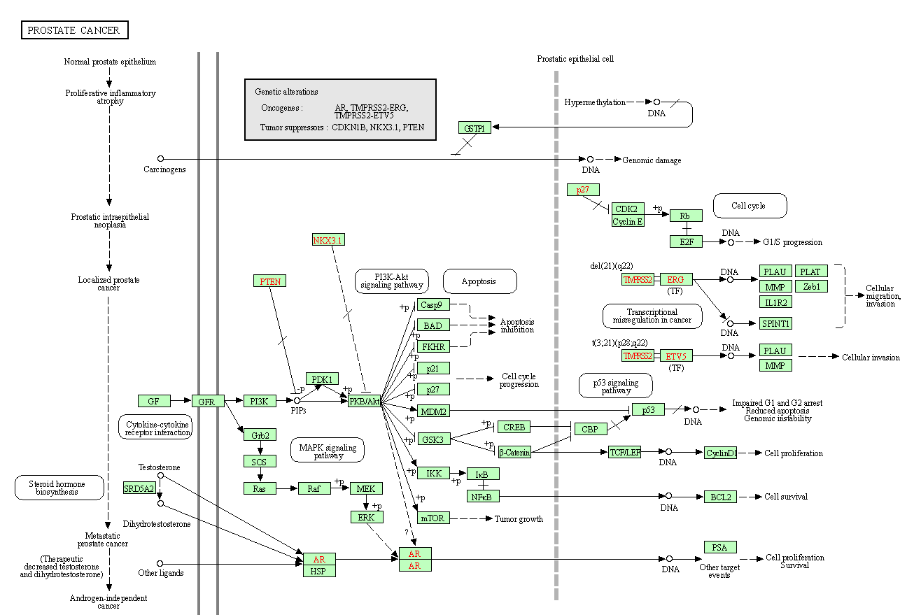

Supplement: Supplementary file 2 — Supplementary Material 2: Figure S2: KEGG pathway analysis of human prostate cancer (ID: hsa05215) illustrating the potential targets and pathways of A. annua chemical constituents where the orange ovals indicated the targets where the molecules interacted and the red rectangles indicated the targeted pathways. [file 12906_2023_4135_MOESM2_ESM.docx]

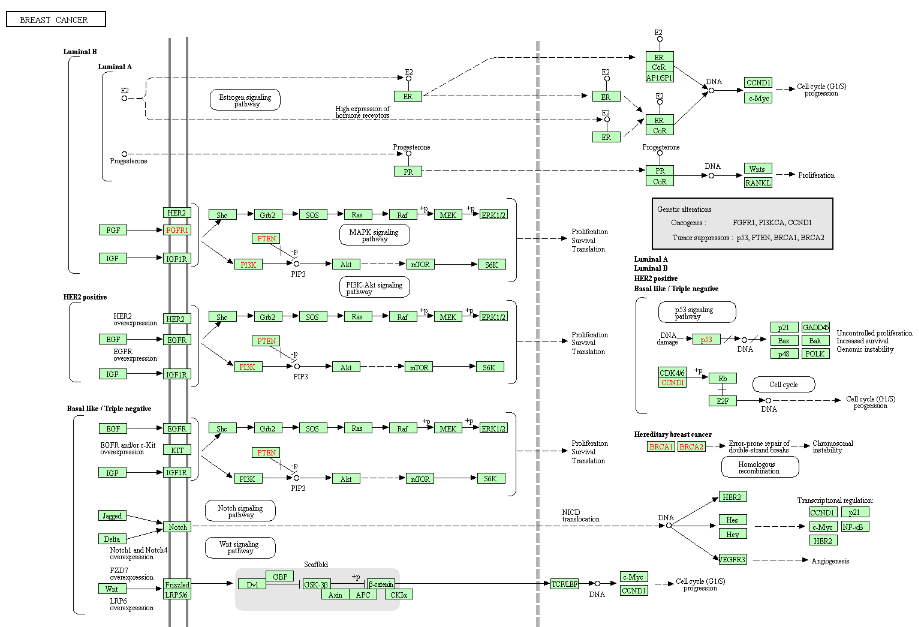

Supplement: Supplementary file 3 — Supplementary Material 3: Figure S3: KEGG pathway analysis of human breast cancer (ID: hsa05224) illustrating the potential targets and pathways of A. annua chemical constituents where the orange ovals indicated the targets where the molecules interacted and the red rectangles indicated the targeted pathways. [file 12906_2023_4135_MOESM3_ESM.docx]

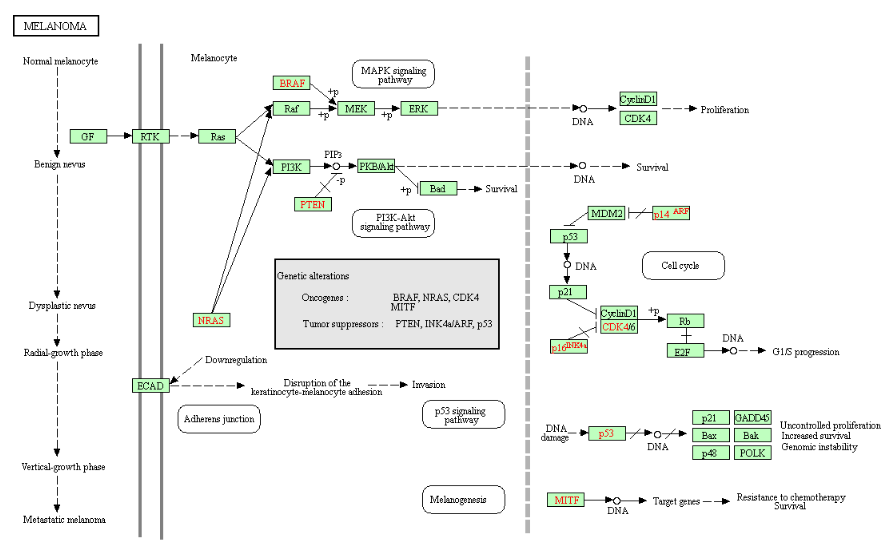

Supplement: Supplementary file 4 — Supplementary Material 4: Figure S4: KEGG pathway analysis of human melanoma (ID: hsa05212) illustrating the potential targets and pathways of A. annua chemical constituents where the orange ovals indicated the targets where the molecules interacted and the red rectangles indicated the targeted pathways. [file 12906_2023_4135_MOESM4_ESM.docx]

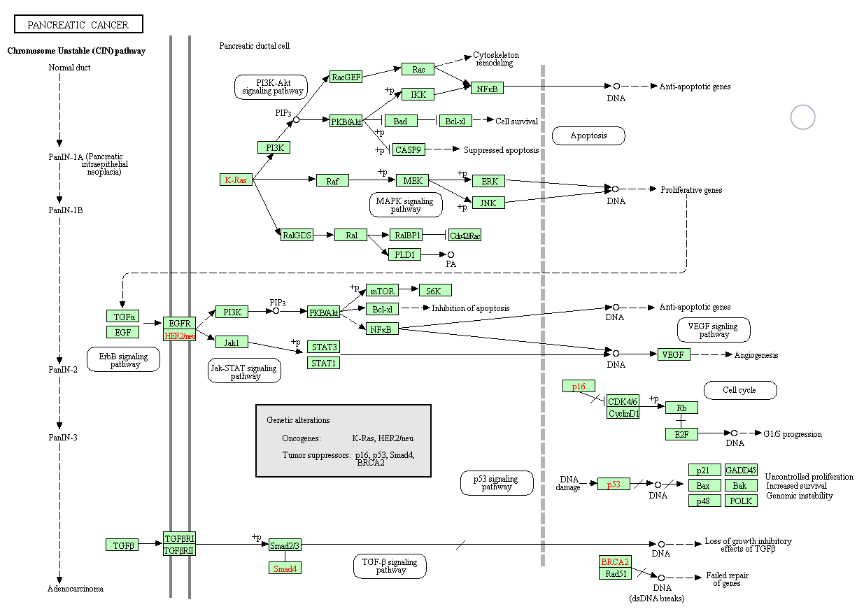

Supplement: Supplementary file 5 — Supplementary Material 5: Figure S5: KEGG pathway analysis of human pancreatic cancer (ID: hsa05218) illustrating the potential targets and pathways of A. annua chemical constituents where the orange ovals indicated the targets where the molecules interacted and the red rectangles indicated the targeted pathways. [file 12906_2023_4135_MOESM5_ESM.docx]
