# Supplementary material for: Computational biology and in vitro studies for anticipating cancer-related molecular targets of sweet wormwood (Artemisia annua)
Source: BMC Complement Med Ther. 2023 Sep 8;23:312. doi: 10.1186/s12906-023-04135-0 (PMC10492370; doi:10.1186/s12906-023-04135-0)
Supplement: Supplementary file 6 — Supplementary Material 6: Video S1: MD Simulation animation of 250 snapshots between 0 and 100 ns of androgen receptor with 4-cadinene-3,11-diol; 3-(2-methylpropanoyl) (PDB ID: 2PIW). Video S2. MD Simulation animation of 250 snapshots between 0 and 100 ns of P53-binding protein with 4-cadinene-3,11-diol; 3-(2-methylpropanoyl) (PDB ID: 4IPF). Video S3. MD Simulation animation of 250 snapshots between 0 and 100 ns of cyclooxygenase-2 with 4-cadinene-3,11-diol; 3-(2-methylpropanoyl) (PDB ID: 3NL1). Video S4. MD Simulation animation of 250 snapshots between 0 and 100 ns of NF-kappa-B with O-(2-Glyceryl)-coniferaldehyde (PDB ID: 1LV2). Video S5. MD Simulation animation of 250 snapshots between 0 and 100 ns of mitogen-activated protein kinase 1 with O-(2-Glyceryl)-coniferaldehyde (PDB ID: 4AN9). Video S6. MD Simulation animation of 250 snapshots between 0 and 100 ns of cyclin-dependent kinase 2 with O-(2-Glyceryl)-coniferaldehyde (PDB ID: 1B39). [file 12906_2023_4135_MOESM6_ESM.pptx]

## Slide 1
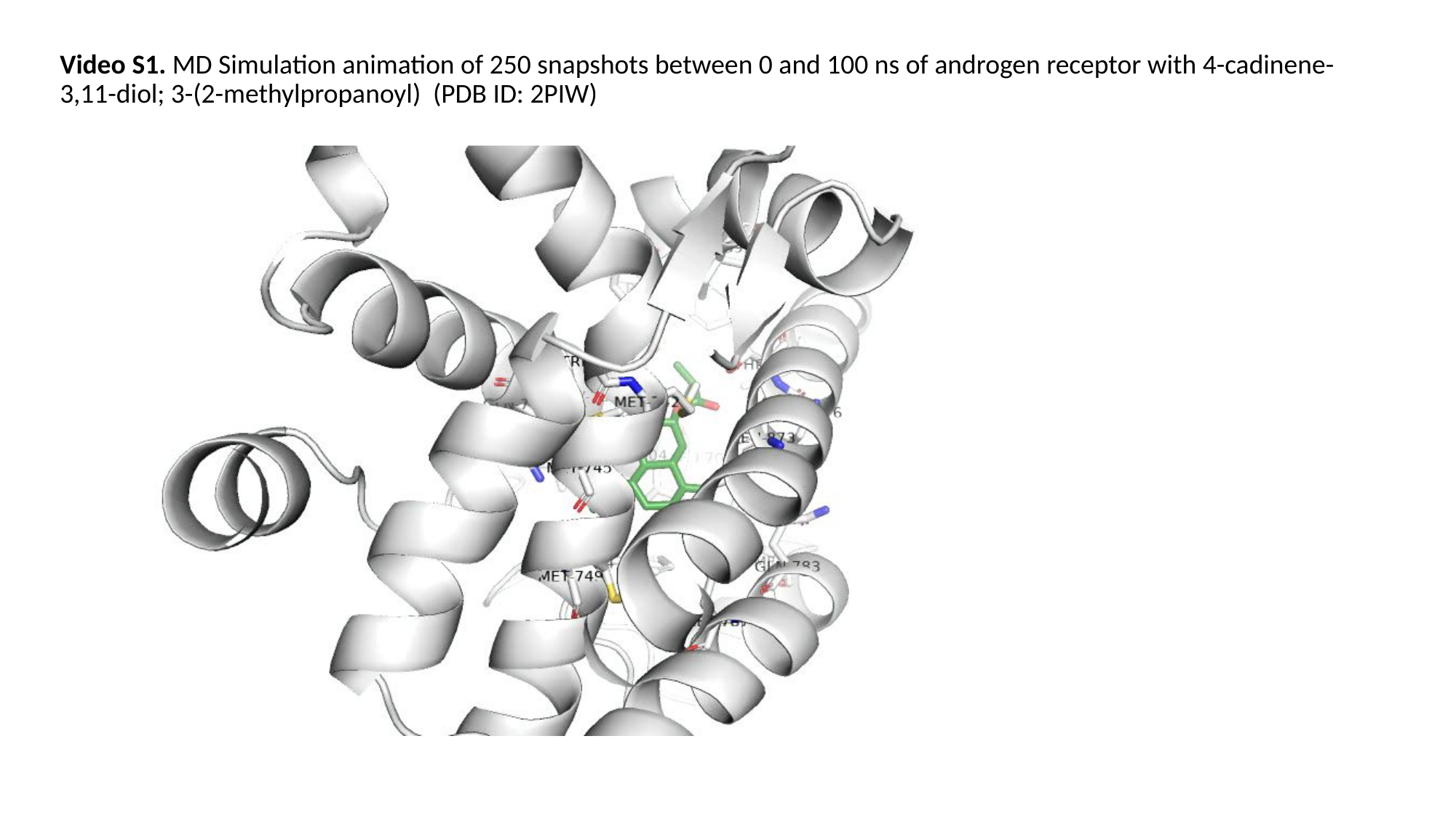

# Video S1. MD Simulation animation of 250 snapshots between 0 and 100 ns of androgen receptor with 4-cadinene-3,11-diol; 3-(2-methylpropanoyl) (PDB ID: 2PIW)

## Slide 2
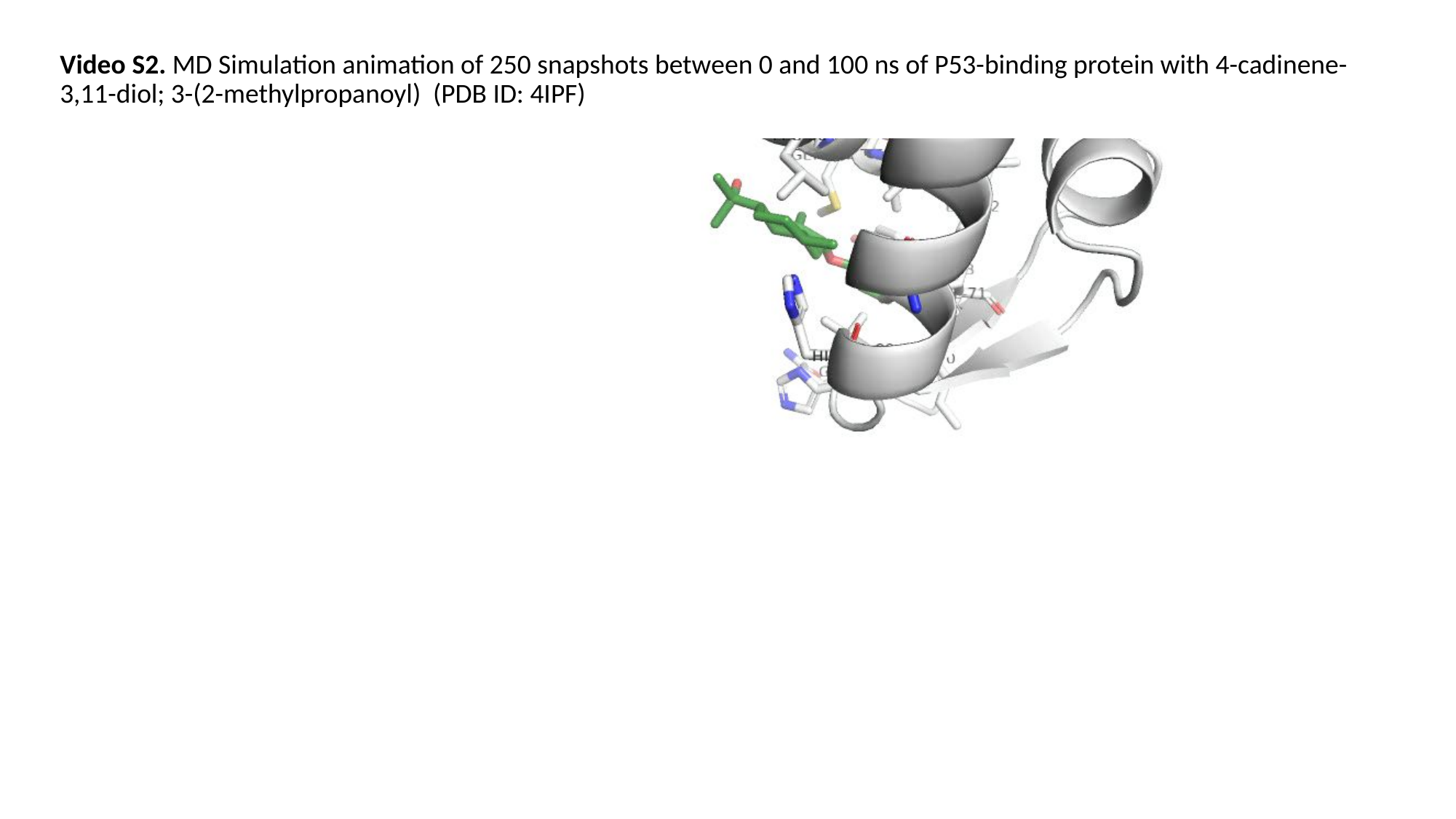

# Video S2. MD Simulation animation of 250 snapshots between 0 and 100 ns of P53-binding protein with 4-cadinene-3,11-diol; 3-(2-methylpropanoyl) (PDB ID: 4IPF)

## Slide 3
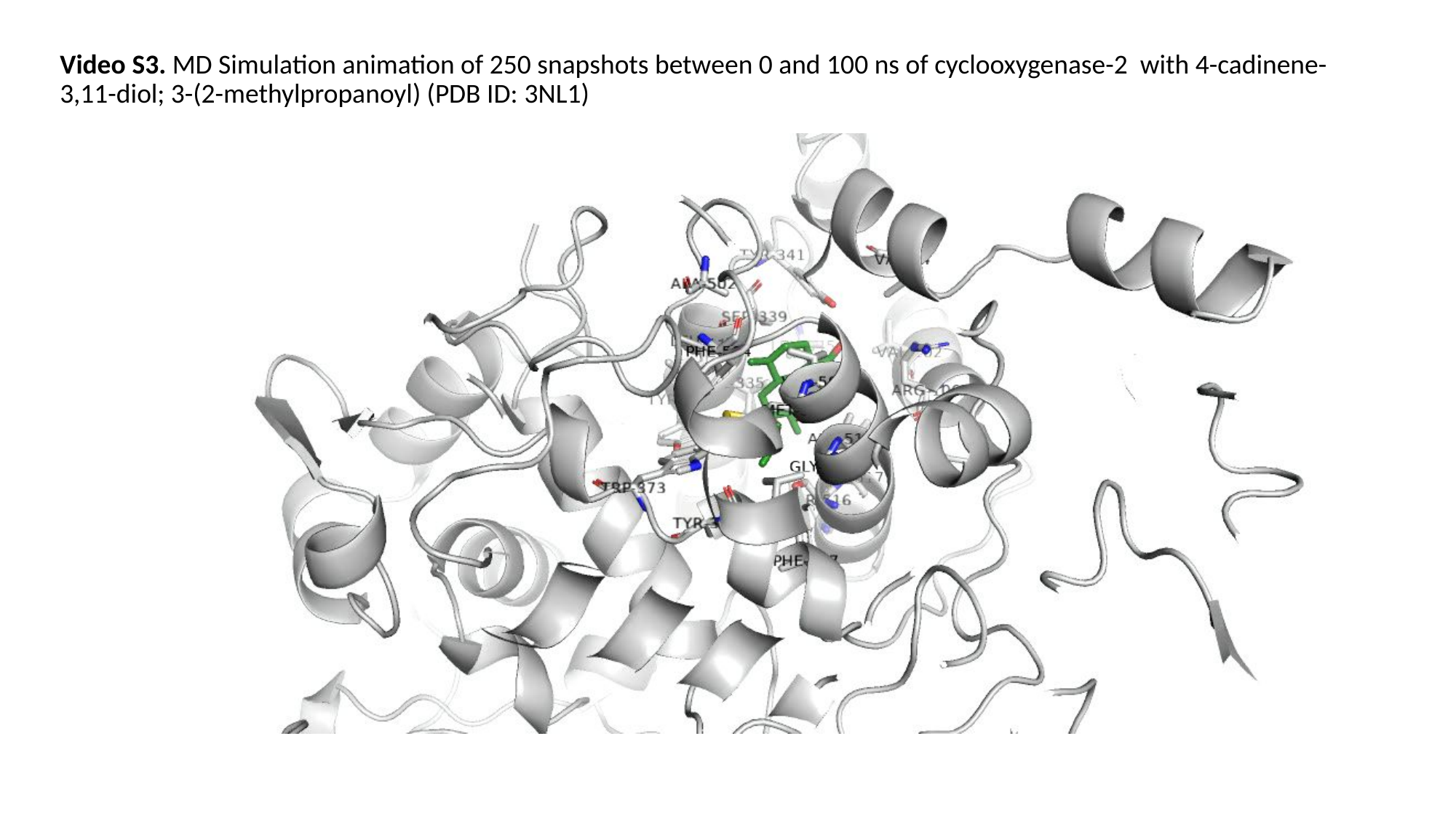

# Video S3. MD Simulation animation of 250 snapshots between 0 and 100 ns of cyclooxygenase-2 with 4-cadinene-3,11-diol; 3-(2-methylpropanoyl) (PDB ID: 3NL1)

## Slide 4
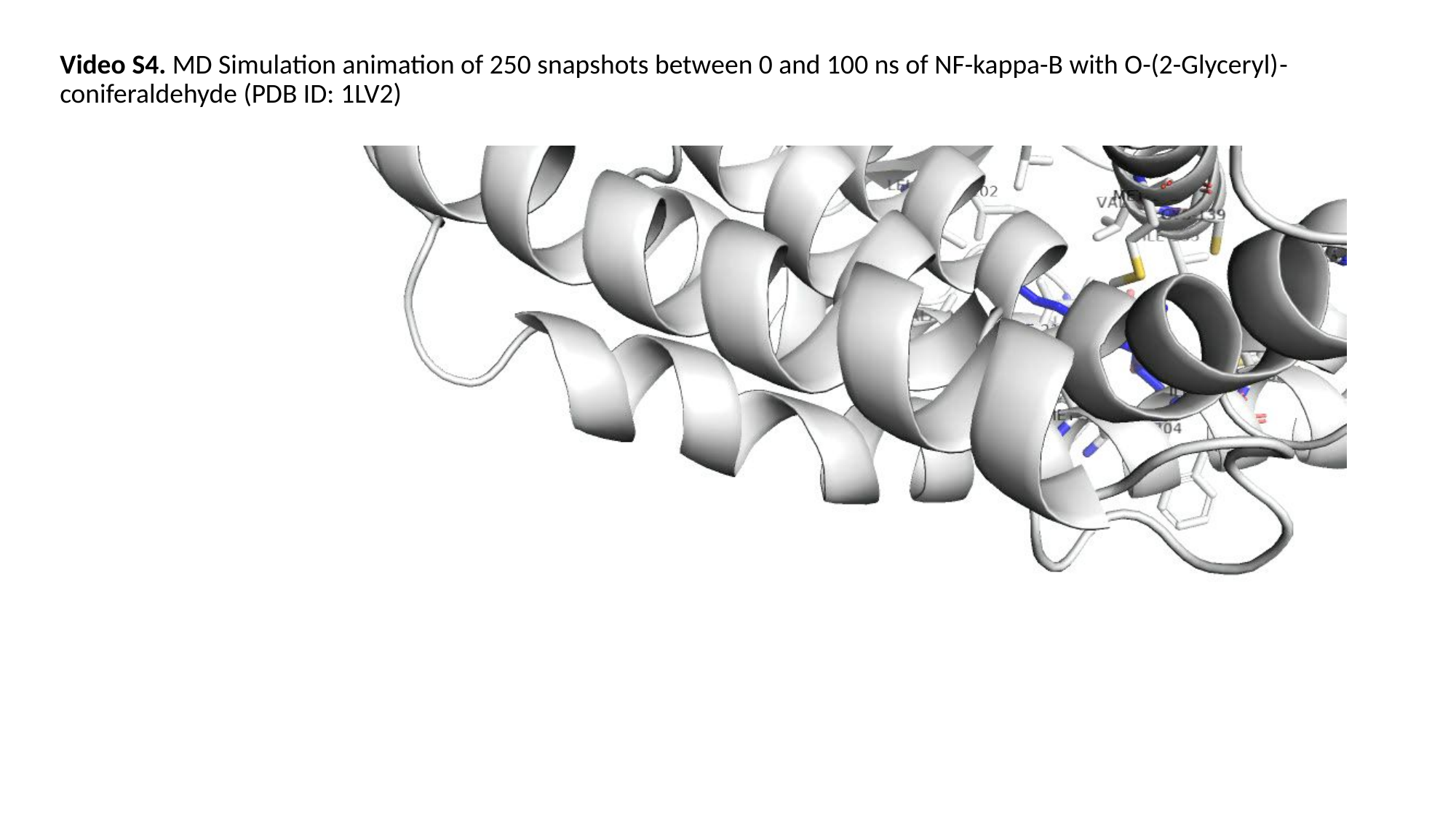

# Video S4. MD Simulation animation of 250 snapshots between 0 and 100 ns of NF-kappa-B with O-(2-Glyceryl)-coniferaldehyde (PDB ID: 1LV2)

## Slide 5
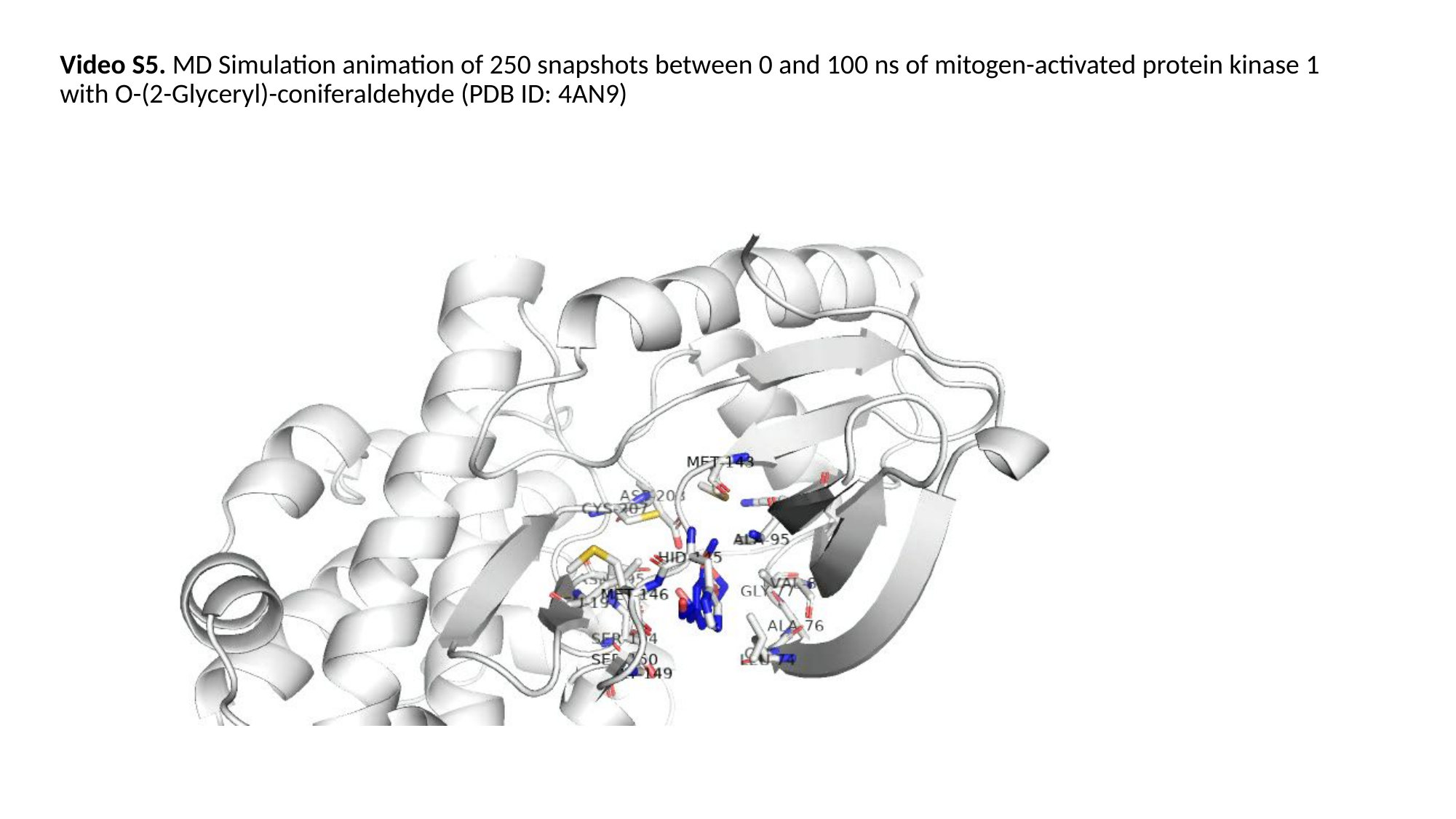

# Video S5. MD Simulation animation of 250 snapshots between 0 and 100 ns of mitogen-activated protein kinase 1 with O-(2-Glyceryl)-coniferaldehyde (PDB ID: 4AN9)

## Slide 6
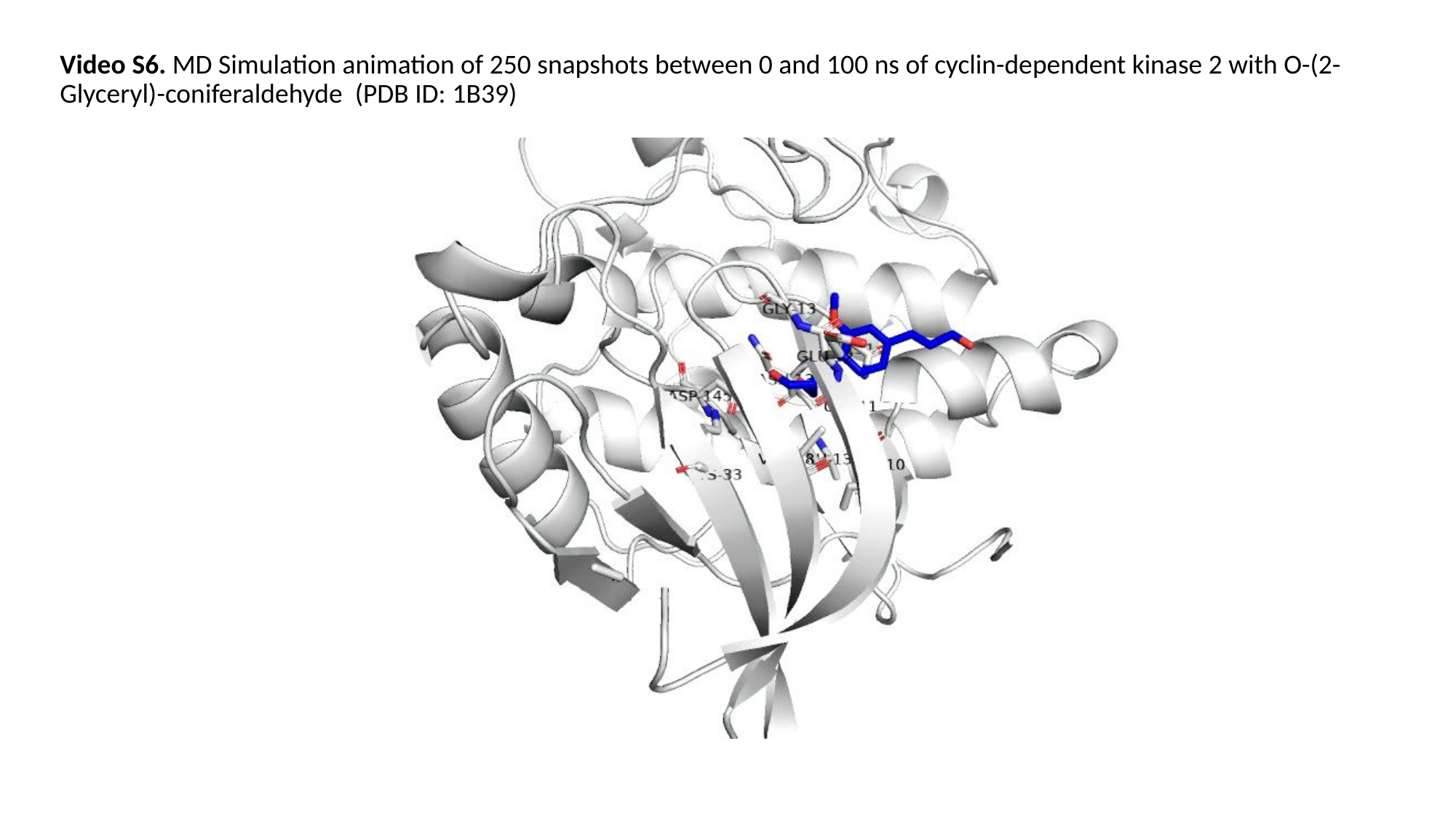

# Video S6. MD Simulation animation of 250 snapshots between 0 and 100 ns of cyclin-dependent kinase 2 with O-(2-Glyceryl)-coniferaldehyde (PDB ID: 1B39)
